# Reversing allosteric communication: From detecting allosteric sites to inducing and tuning targeted allosteric response

**DOI:** 10.1371/journal.pcbi.1006228

**Published:** 2018-06-18

**Authors:** Wei-Ven Tee, Enrico Guarnera, Igor N. Berezovsky

**Affiliations:** 1 Bioinformatics Institute (BII), Agency for Science, Technology and Research (A*STAR), Matrix, Singapore; 2 Department of Biological Sciences (DBS), National University of Singapore (NUS), Singapore; Danish Cancer Society Research Center, DENMARK

## Abstract

The omnipresence of allosteric regulation together with the fundamental role of structural dynamics in this phenomenon have initiated a great interest to the detection of regulatory exosites and design of corresponding effectors. However, despite a general consensus on the key role of dynamics most of the earlier efforts on the prediction of allosteric sites are heavily crippled by the static nature of the underlying methods, which are either structure-based approaches seeking for deep surface pockets typical for “traditional” orthosteric drugs or sequence-based techniques exploiting the conservation of protein sequences. Because of the critical role of global protein dynamics in allosteric signaling, we investigate the hypothesis of reversibility in allosteric communication, according to which allosteric sites can be detected via the perturbation of the functional sites. The reversibility is tested here using our structure-based perturbation model of allostery, which allows one to analyze the causality and energetics of allosteric communication. We validate the “reverse perturbation” hypothesis and its predictive power on a set of classical allosteric proteins, then, on the independent extended benchmark set. We also show that, in addition to known allosteric sites, the perturbation of the functional sites unravels rather extended protein regions, which can host latent regulatory exosites. These protein parts that are dynamically coupled with functional sites can also be used for inducing and tuning allosteric communication, and an exhaustive exploration of the per-residue contributions to allosteric effects can eventually lead to the optimal modulation of protein activity. The site-effector interactions necessary for a specific mode and level of allosteric communication can be fine-tuned by adjusting the site’s structure to an available effector molecule and by the design or selection of an appropriate ligand.

## Author summary

Recent advances in the development of allosteric drugs allow one to fully appreciate the sheer power of allosteric effectors in the avoiding toxicity, receptor desensitization and modulatory rather than on/off mode of action, compared to the traditional orthosteric compounds. The detection of allosteric sites is one of the major challenges in the quest for allosteric drugs. This work proposes a “reverse perturbation” approach for identifying allosteric sites as a result of a perturbation applied to the functional ones. We show that according to the traditional Monod-Changeux-Jacob’s definition of allostery, considering non-overlapping regulatory and functional sites is a critical prerequisite for the successful detection of allosteric sites. Using the reverse perturbation method, it is possible to determine wide protein regions with a potential to induce an allosteric response and to adjust its strength. Further studies on inducing and fine-tuning of allosteric signalling seem to be of a great importance for efficient design of non-orthosteric ligands in the development of novel drugs.

## Introduction

The traditional emphasis on complementarity between the drug and the catalytic site has inarguably formed a foundation in the current drug discovery approaches. However, many important drug targets share a conserved substrate binding site [1–5], rendering drug toxicity as a result of the off-target binding. The allosteric regulation of protein activity via effector binding has been increasingly favoured in the drug discovery [1, 2, 6]. It is well recognized that potentially druggable allosteric sites are ubiquitous in most if not all dynamic proteins [7], turning the key advantage of targeting allosteric sites [5]—non-competitive fine-tuning of protein activity at a distance—into a new paradigm in the drug design. For example, it was shown that allosteric drugs provide a way to modulating the activity of kinases that underlie a multitude of human diseases, bypassing the problem of low specificity with the conserved ATP binding pocket [8, 9]. The allosteric drugs for GPCRs provide greater subtype selectivity among GPCR receptor families, while avoiding receptor desensitization typical for the orthosteric ones [10–12]. Currently, among the notable marketed drugs targeting GPCRs are cinacalcet [13] and maraviroc [14], an allosteric agonist for calcium-sensing receptor and an antiretroviral allosteric antagonist for the CCR5 receptor, respectively.

One of the major hurdles in the development of allosteric drugs lies in the finding of allosteric sites [5, 15–17], for which a repertoire of experimental and computational methods is being developed. High-throughput fragment-based screening using a large chemical library formed the main thrust in the identification of potential allosteric sites and lead compounds in pharmaceutical research [18–20]. A number of site-directed approaches have been employed for detection and probing of allosteric sites and their modulatory effects, including disulfide trapping [21], alanine scanning [22], hydrogen-deuterium exchange mass spectrometry [23, 24] and photoaffinity [25, 26]. However, above experimental approaches while powerful, are relatively costly and time-consuming compared to any extensive analysis performed *in silico*. Computational approaches for finding the allosteric sites can be broadly classified as sequence-based and structure-based methods [3, 4, 27]. Sequence-based techniques utilize sequence homology inferred from the multiple sequence alignment to identify the co-evolving amino acids that constitute catalytic and allosteric sites [28]. However, complexity of the site-ligand interactions and energetics result in strong limitations on the predictive power of the sequence-based approaches [5, 28, 29]. Structure-based methods analyse binding pockets based on their topological and physicochemical features [5, 30]. These approaches are strongly biased towards binding pockets that exhibit detectable curvature in the static 3D structure, in which case latent allosteric sites that can exist in a subset of a protein conformational ensemble may be left undetected.

We have recently introduced a structure-based perturbation model of allostery [31], which quantitatively describes the causality and energetics of allosteric communication, by simulating ligand binding as a local alteration in the protein inter-residue network of interactions. Because of the observation that perturbation at allosteric sites can affect distant functional site via modifying the energetics of the whole protein and assuming the reversibility of allosteric signalling, we hypothesized here that allosteric sites could be detected by perturbing the functional ones [5]. In order to test this hypothesis and to explore its predictive power, the reverse perturbation approach was developed here. Using a heterogeneous set of 13 classical allosteric proteins from previous studies [31–33], dubbed here the “classical set”, we found that perturbation at the functional sites allows one to identify known allosteric ones. In order to estimate the predictive power of reverse perturbation method, it was necessary to introduce an operational definition of the allosteric site in the framework of elastic network model of protein. Specifically, assuming that allosteric signalling occurs between non-overlapping distant sites, a distance condition was set to ensure communication and not direct physical interaction between residues of the functional and allosteric sites. Using the classical set and a new collection of 41 allosteric proteins from the benchmarking set [34], predictive power of the reverse perturbation method is shown. Furthermore, we argue that in addition to the widely addressed case of predicting latent allosteric sites [35], the task of inducing allosteric signalling with a desired and tunable level of agonistic/antagonistic activity can be naturally formulated. We show that the reverse perturbation approach opens the way for achieving above goal, allowing one to find targets for allosteric effectors and to optimize structures/compositions and interactions of corresponding regulatory site-effector pairs that will provide a desired allosteric response at the functional site.

## Results

### Identification of allosteric sites by the reverse perturbation approach

**Fig 1:**
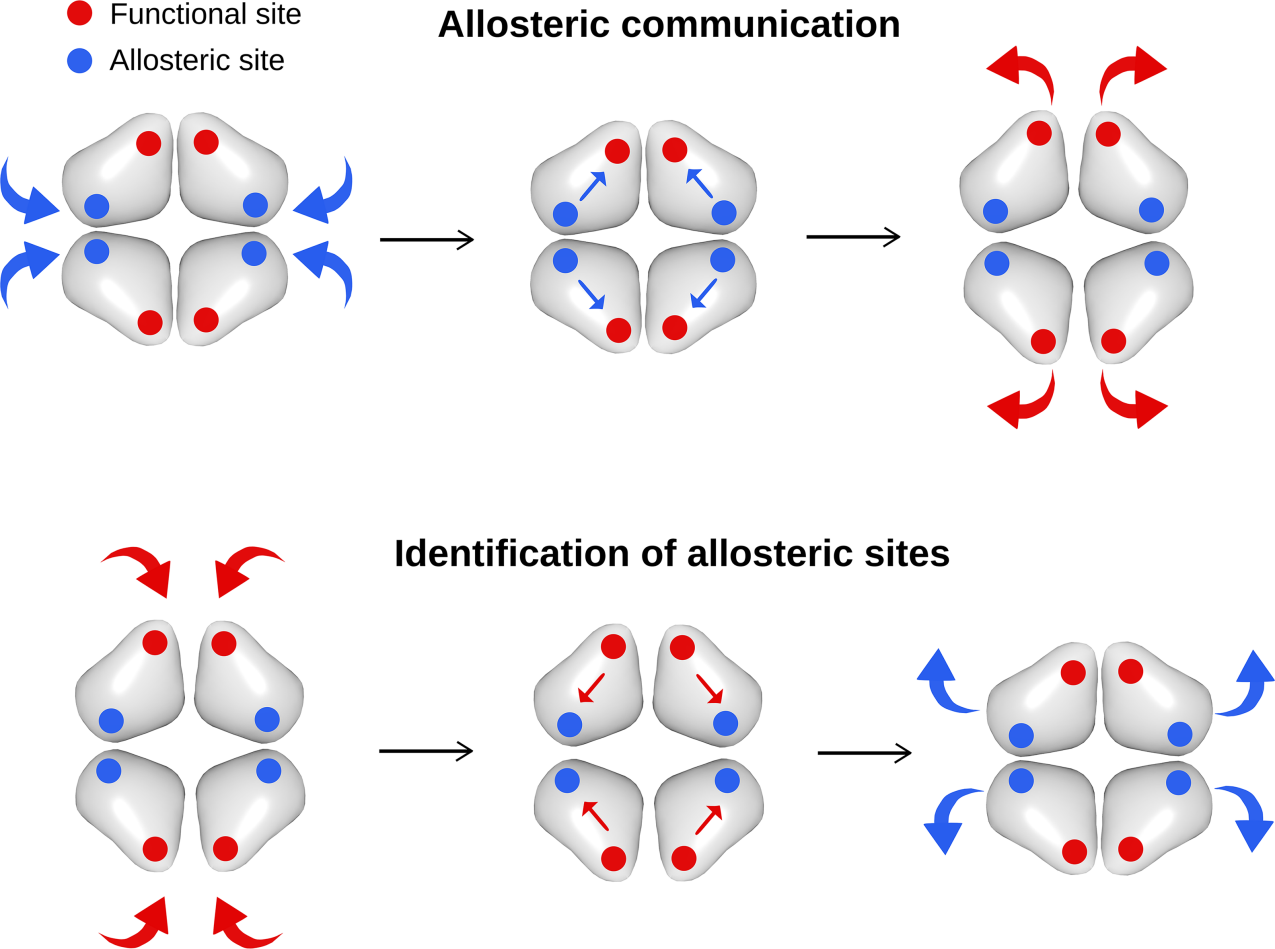
Reversing the allosteric communication leads to the identification of allosteric sites. In allosteric communication, binding of effectors at allosteric sites causes a change of configurational work exerted in the distant functional sites. In the hypothesis of reversibility of allosteric communication, simulated ligand binding at the functional sites enables the identification of allosteric sites.

In the structure-based statistical mechanical model of allostery [31], ligand binding is modelled as a perturbation of the harmonic network associated with the protein. The perturbation is defined as a stiffening harmonic restraint applied to the residue pairs that compose the binding site of interest. As a result of the perturbation, residues in the binding site experience an increase of rigidity in comparison with the residues in the unperturbed binding site. We have shown that in the event of allosteric communication, the perturbation of the allosteric sites induces a response at the functional ones by altering their energetics and fluctuation dynamics [31]. Specifically, as a result of the perturbation in a binding site, a per-residue free energy change Δ*g*_*i*_ is obtained for each residue of the protein, which is the signature of the change in the amount of work exerted in the environment of residue *i*. Here we investigate the hypothesis of reversibility of allosteric communication, according to which one can detect a change in the free energy on the residues of allosteric sites when a perturbation–simulated binding–is applied in the functional sites (Fig 1).

To begin with, we analysed a set of classical allosteric proteins, dubbed here “classical set” [32, 33], to directly test the hypothesis of the reverse perturbation approach in identifying allosteric sites in proteins of different sizes, oligomerization states and functions. Table 1 contains the average free energy changes (averaged over all residues in corresponding sites in case of oligomeric proteins) observed as a result of the allosteric signalling in known allosteric sites (Δ*g*_*A*_) and in the restrained functional sites (Δ*g*_*F*_) in 13 proteins of the classical set.

**Table 1:** Free energy changes at the functional and allosteric sites upon reverse perturbation of the allosteric communication for the proteins of the classical set.

Protein (#chain, #res)	PDB ID	Perturbation at functional site	Δ*g*_*F*_ (*kcal/mol*)	Allosteric Site (A/I)	Δ*g*_*A*_ (*kcal/mol*)	Δ*g*_*U*_ (*kcal/mol*)	Proximity (%)
AnthS (2+2, 1426)	1i7q	2 × GLU	-0.83	TRP (I)	0.42	-0.29	0
ATCase (2×3+3×2, 2736)	1d09	6 × PAL	-1.08	ATP-CTP (A/I)	1.75	0.22	0
BGDH (6, 2976)	1nr7	6 × GLU/NDP	-0.52 -1.17	ADP (A) GTP (I)	0.18 -0.18	0.31	3 7
CAP (2, 418)	1o3q	2 × DNA	-1.11	cAMP (A)	1.73	0.71	0
DAHPS (4, 1401)	1kfl	4 × PEP	-0.19	PHE (I)	0.80	0.15	1
DAK (2, 735)	3ju5	1 × ATP/ARG	-1.92 -0.75	ATP (I) ARG (I)	0.48 1.01	-0.01	0
G6PD (6, 1596)	1cd5	6 × AGP	0.36	16G (A)	0.78	0.66	10
NADME (4, 2232)	1efk	4 × NAD	-0.82	FUM (A)	2.90	0.67	0
PFK (4, 1284)	3pfk	4 × F6P/ADP	0.43 -0.63	PEP (I) ADP (A)	1.47 1.35	0.33	0 2
PGDH (4, 1624)	1yba	4 × AKG/NAD	-0.77 -1.94	SER (I)	2.62	0.01	0
PTP1B (1,278)	2hnp	1 × BPM	-3.36	892 (I)	0.42	-2.12	0
SSUPRT (4, 868)	1xtu	4 × UMP	-0.73	CTP (I)	2.05	-0.05	1
ThrS (2, 884)	1e5x	2 × PLP	-3.06	SAM (A)	2.27	0.05	0

First column: Protein name, oligomerization state, and total number of residues. Second column: PDB ID of the protein. The third and fourth columns represent the perturbation applied to functional sites and the resulted Δ*g*_*F*_ values, respectively (see Eq 7 in Materials and Methods). The fifth and sixth columns show the known allosteric sites (A—allosteric activator, I—allosteric inhibitor) and the Δ*g*_*A*_ values in the allosteric sites in response to the reverse perturbation, respectively. The seventh column provides the average free energy differences Δ*g*_*U*_ over all the residues in the protein, giving an estimate on the protein stability changes caused by the applied perturbation. The last column gives the proximity between the corresponding functional and allosteric sites in a protein subunit (see Eq 8 in Materials and Methods).

### The set of classical allosteric proteins

**Fig 2:**
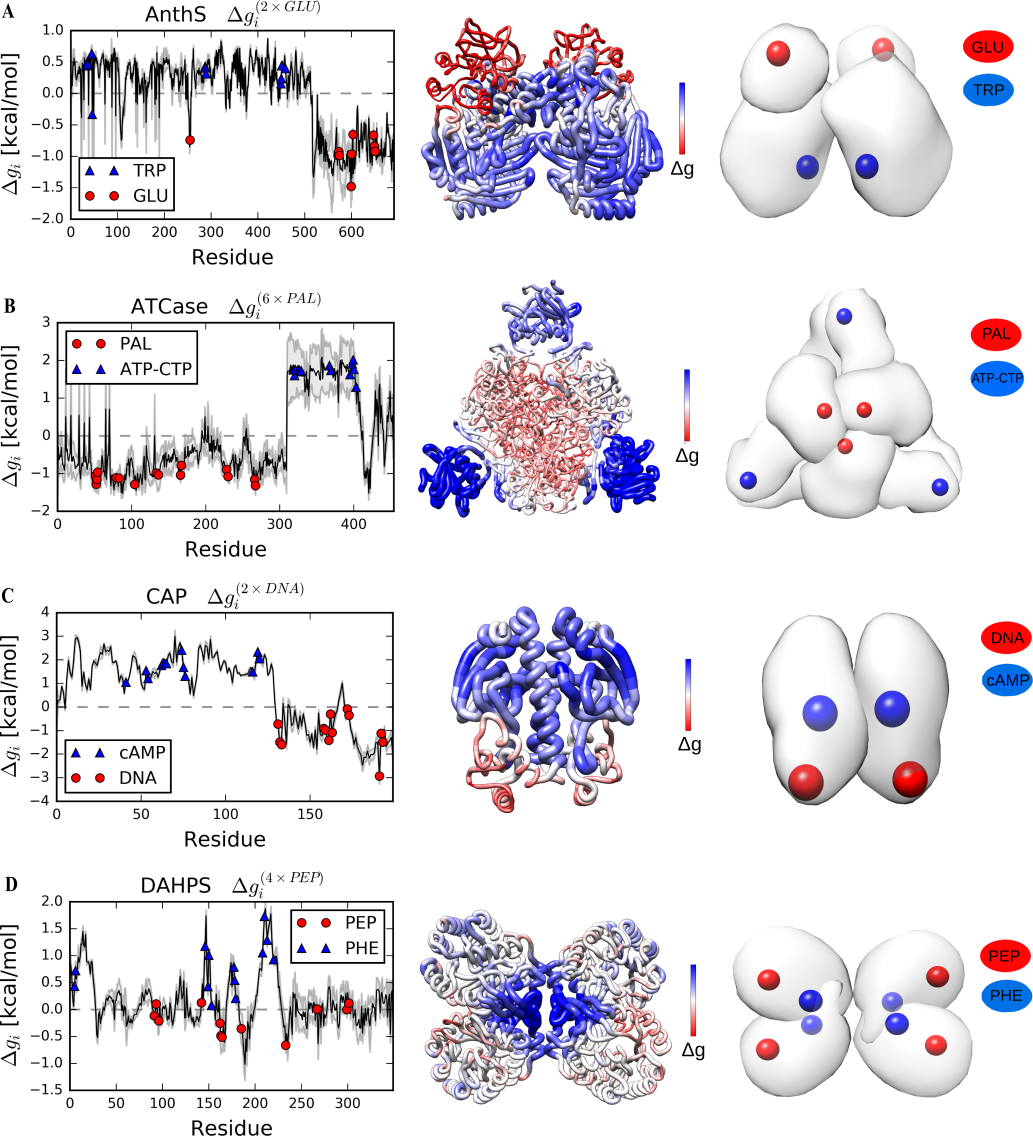
Free energy profiles obtained as a result of the reverse perturbation for AnthS, ATCase, CAP and DAHPS. (A) AnthS, a heterotetramer involved in the biosynthesis of anthranilate. (B) ATCase, a heterododecamer that catalyzes the first step in pyrimidine biosynthesis. (C) CAP, a homodimeric transcriptional activator. (D) DAHPS, a homotetramer involved in the biosynthesis of aromatic amino acids. The free energy changes are the average from corresponding residues in different subunits. The symbols indicate residues in corresponding ligand binding sites. Grey error bands indicate the standard deviation of Δ*g*_*i*_ across the subunits. In the middle, the structure of the protein in the C_α_ coarse-grained model is colored according to the free energy change (increased–blue, decreased–red). The tube radius is proportional to the value of the free energy change. Colored spheres indicate ligand binding sites in a simplified quaternary structure depicted on the right: red or orange colors are used for functional sites, blue–for allosteric sites.

The Anthranilate synthase (AnthS) from *Serratia marcescens* is a heterotetramer consisting of a dimer of TrpE and TrpG subunits. Upon perturbation of the glutamine substrate binding sites, the tryptophan (inhibitor) binding sites in the larger TrpE subunits show a positive free energy difference response ($\Delta g_{TRP}^{\left(2\times GLU\right)}$ = 0.42 kcal/mol), compared to the overall decrease in the free energy of the structure and stabilization of the entire TrpG subunits upon perturbation ($\Delta g_{AnthS}^{\left(2\times GLU\right)}$ = -0.29 kcal/mol, Fig 2A and Table 1).

The aspartate carbamoyltransferase (ATCase, Fig 2B) from *Escherichia coli* is a heterododecameric enzyme composed of two trimers of catalytic subunits in the centre of the oligomer and three dimers of the peripheral regulatory subunits. Simulated binding in the catalytic sites of ATCase (PAL sites) increases the configurational work exerted at the allosteric domain in the regulatory subunits that contain allosteric activator ATP and inhibitor CTP ($\Delta g_{ATP-CTP}^{\left(6\times PAL\right)}$ = 1.75 kcal/mol, Table 1), but not the zinc-binding domain which plays a structural role in the complex assembly.

Allosterically activated by cyclic AMP, the catabolite activator protein (CAP) from *E*. *coli* is a classical model system of transcriptional activation. The cAMP binding causes large rotation in the DNA-binding domain of CAP, which is a prerequisite for interactions with DNA [36]. Negative cooperativity observed for the binding of second cAMP molecule was discussed earlier [31], and it was shown that mutations in the cAMP-binding pocket decreasing the affinity between CAP and cAMP enhance negative cooperativity [37]. Using the DNA-bound conformation of CAP, we simulated binding in the DNA interaction sites and observed that allosteric site in the N-terminal cAMP-binding domains exhibits a large positive free energy change ($\Delta g_{cAMP}^{\left(2\times DNA\right)}$ = 1.73 kcal/mol), higher than the average free energy change ($\Delta g_{CAP}^{\left(2\times DNA\right)}$ = 0.71 kcal/mol) of the homodimer (Fig 2C and Table 1).

For the homotetrameric 3-deoxy-D-arabino-heptulosonate-7-phosphate synthase (DAHPS) from *E*.*coli*, binding of the phosphoenolpyruvate (PEP) substrate to the TIM barrel domain was simulated. We observed a positive free energy change ($\Delta g_{PHE}^{\left(4\times PEP\right)}$ = 0.80 kcal/mol) in the center of the quaternary complex, where the inter-subunit pockets for allosteric inhibitor phenylalanine (PHE) are located, while the whole structure yields rather a modest positive free energy change ($\Delta g_{DAHPS}^{\left(4\times PHE\right)}$ = 0.15 kcal/mol, Fig 2D and Table 1).

**Fig 3:**
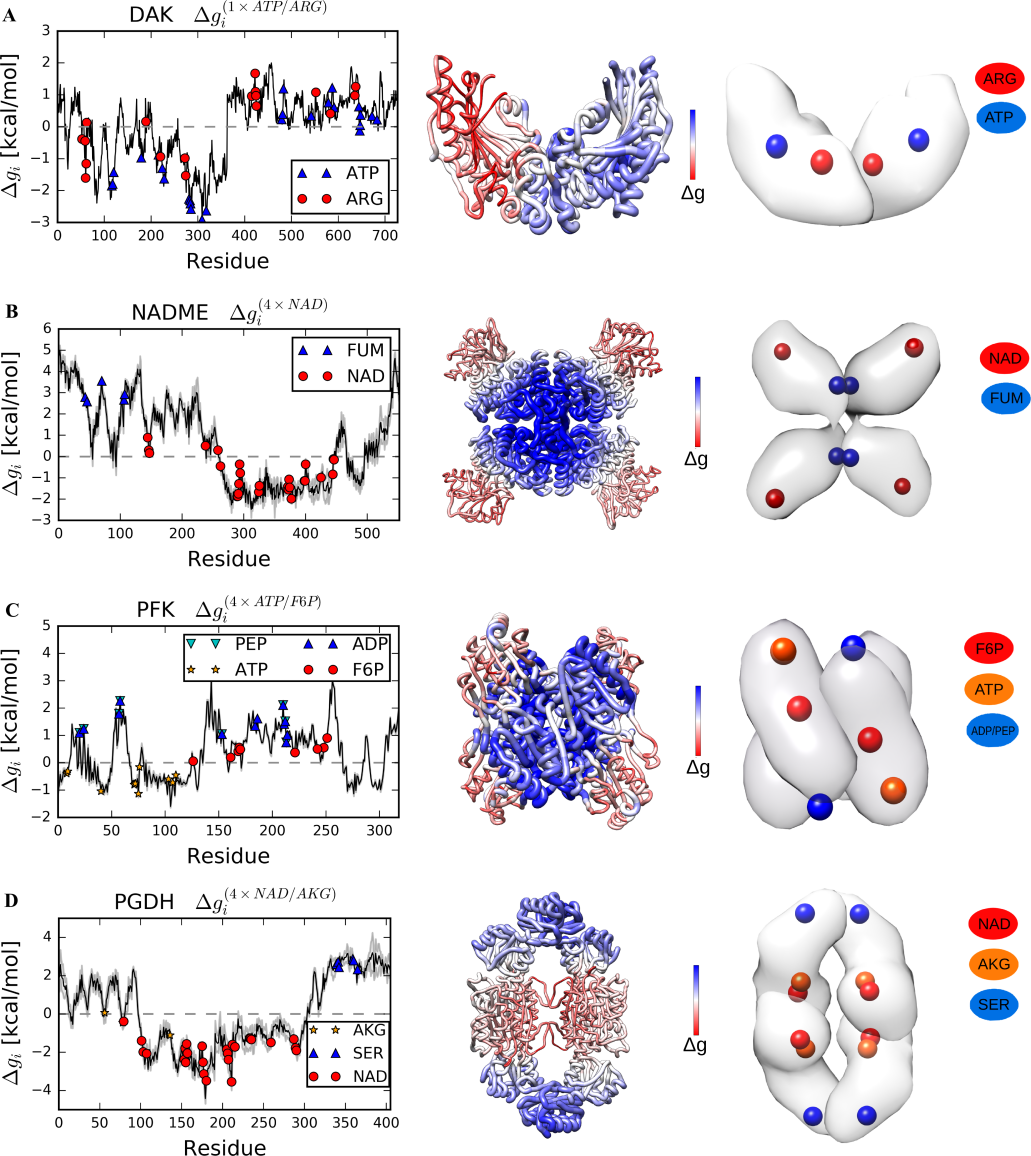
Free energy profiles obtained as a result of the reverse perturbation for DAK, NADME, PFK and PGDH. (A) DAK, a dimeric arginine kinase. (B) NADME, a homotetramer for oxidative decarboxylation of malate to pyruvate. (C) PFK, the homotetramer is a key enzyme in glycolytic pathway. (D) PGDH, a homotetramer involved in the biosynthesis of serine.

The dimeric arginine kinase (DAK) from *Apostichopus japonicus* is known to exhibit negative cooperativity, in which binding of substrates (ARG and ATP) to one subunit causes a conformational change in the free subunit. Specifically, “large outward reorganization of the other subunit (open state) which result in the release of products”, that precludes substrate binding was experimentally observed [38]. We found that perturbing the catalytic sites in one subunit destabilizes the unperturbed one ($\Delta g_{ARG}^{\left(1\times ARG/ATP\right)}$ = 1.01 kcal/mol and $\Delta g_{ATP}^{\left(1\times ARG/ATP\right)}$ = 0.48 kcal/mol, Fig 3A and Table 1), presumably reducing its affinity to substrates.

The NAD binding sites in the Rossmann fold were perturbed in the human NAD-dependent malic enzyme (NADME). Fig 3B shows that configurational work propagates from the outer domains of the homotetramer to the dimerization interface where the allosteric activator fumarate (FUM) binds, increasing the amount of work exerted in these sites ($\Delta g_{FUM}^{\left(4\times NAD\right)}$ = 2.90 kcal/mol) to a much larger extent than the overall free energy change ($\Delta g_{NADME}^{\left(4\times NAD\right)}$ = 0.67 kcal/mol, Fig 3B and Table 1). Noteworthy, latent allosteric sites could also exist in the extended area near the core of this quaternary complex where large positive configurational work is observed as a result of perturbation at the functional sites.

A classical allosteric enzyme with a long history of studies, the phosphofructokinase (PFK) from *Bacillus stearothermophilus*, is a homotetramer. When the substrate and cofactor binding sites for fructose-6-phosphate (F6P) and ATP were perturbed, the allosteric response at the regulatory exosites (that bind activator ADP and inhibitor phosphoenolpyruvate (PEP)) located in the dimerization interface is manifested in the increase of the free energy ($\Delta g_{ADP}^{\left(4\times F6P/ATP\right)}$ = 1.35 kcal/mol and $\Delta g_{PEP}^{\left(4\times F6P/ATP\right)}$ = 1.47 kcal/mol) detected in these sites, respectively (Fig 3C and Table 1).

The D-3-phosphoglycerate dehydrogenase (PGDH) of *E*. *coli* is a homotetramer with a ring-shaped quaternary structure. Simulated binding at the substrate AKG and cofactor NAD sites, which are located in the interface between corresponding substrate and cofactor domains, allows one to identify the binding sites for allosteric inhibitor serine (SER) in the distant peripheral regulatory domains. The serine binding sites show a large positive free energy change ($\Delta g_{SER}^{\left(4\times AKG/NAD\right)}$ = 2.62 kcal/mol) in comparison with the negligible background free energy increase (Fig 3D and Table 1).

**Fig 4:**
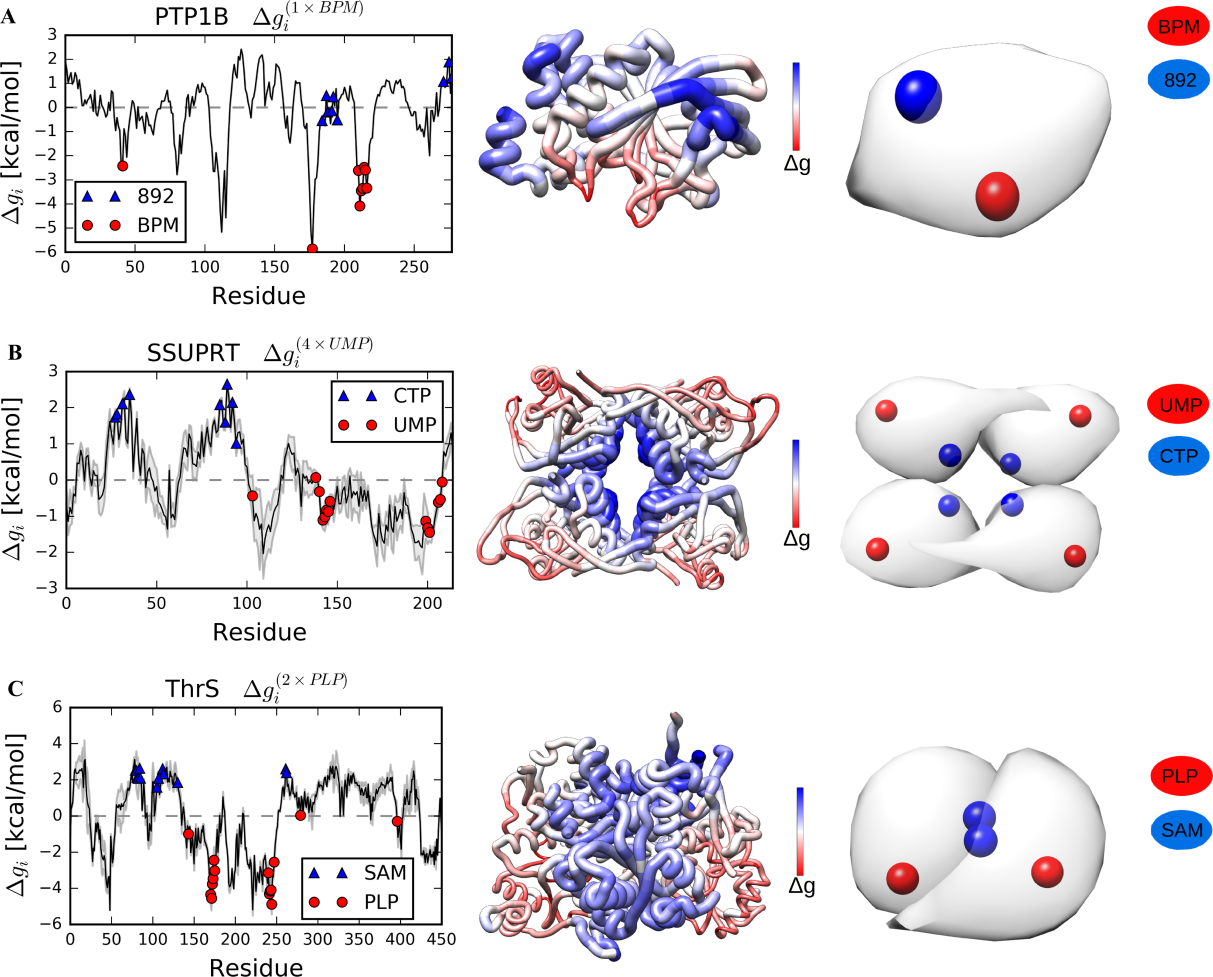
Free energy profiles obtained as a result of the reverse perturbation for PTP1B, SSUPRT and ThrS. (A) PTP1B, a monomeric enzyme that regulates the insulin signalling pathway. (B) SSUPRT, a homotetramer that catalyzes the synthesis of uridine monophosphate. (C) ThrS, a homodimer for the biosynthesis of threonine.

The overall change in the free energy of the small monomeric human protein tyrosine phosphatase 1B (PTP1B) is negative upon restraining the catalytic site BPM ($\Delta g_{PTP1B}^{\left(1\times BPM\right)}$ = -2.12 kcal/mol), pointing to the strong stabilizing role of this perturbation. We observed a slight increase of the free energy at the known allosteric site 892 ($\Delta g_{892}^{\left(1\times BPM\right)}$ = 0.42 kcal/mol, Fig 4A and Table 1). Additionally, the beta sheet distant from the catalytic site exhibited large increase in the free energy change, suggesting the presence of a potential latent allosteric site (Fig 4A).

The uracil phosphoribosyltransferase from *Sulfolobus solfataricus* (SSUPRT) is a homotetramer that catalyzes the production of uridine 5′-monophosphate (UMP). As a result of perturbing the catalytic sites UMP in the outer regions (Fig 4B), residues in the binding site for the allosteric inhibitor CTP at the central interface show the positive free energy change ($\Delta g_{CTP}^{\left(4\times UMP\right)}$ = 2.05 kcal/mol, Table 1) compared to the overall small average free energy change ($\Delta g_{SSUPRT}^{\left(4\times UMP\right)}$ = -0.05 kcal/mol).

The homodimer of threonine synthase (ThrS) from *Arabidopsis thaliana* features an extensive interface between the subunits, in which the allosteric activator S-adenosylmethionine (SAM) binds. Similar to SSUPRT, when the protein’s catalytic sites pyridoxal-L-phosphate PLP are stabilized, the configurational work exerted on the binding sites for SAM increases ($\Delta g_{SAM}^{\left(2\times PLP\right)}$ = 2.27 kcal/mol, Table 1) in contrast to the small average free energy change ($\Delta g_{ThrS}^{\left(2\times PLP\right)}$ = 0.05 kcal/mol, Fig 4C).

**Fig 5:**
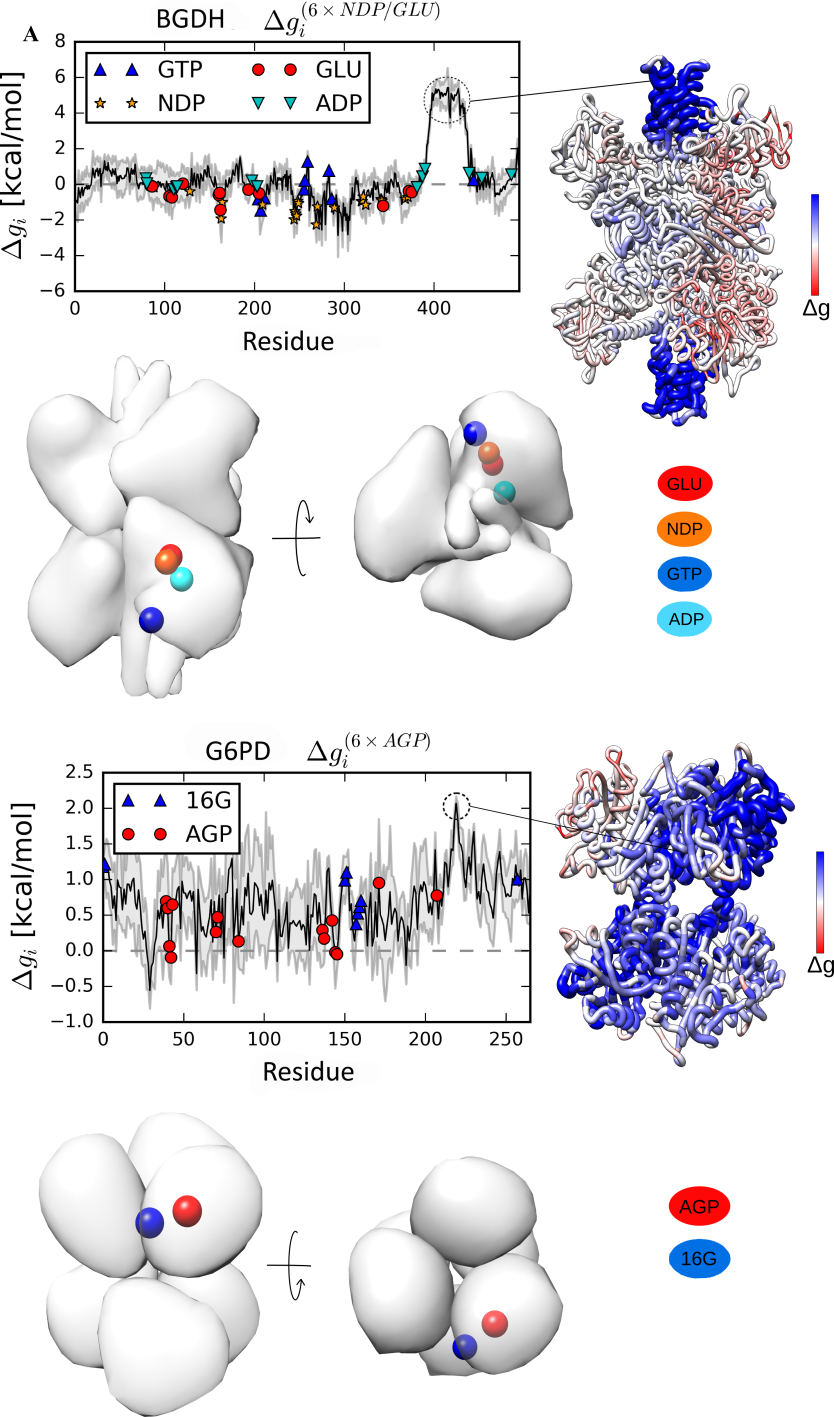
Results of the reverse perturbation of allosteric communication in the large BGDH and G6PD homohexamers. Reverse perturbation of the allosteric communication suggested the presence of latent allosteric sites in the antenna region of the BGDH (A) and various locations of the G6PD (B). The antenna region of BGDH and the Cys219 in the core of G6PD are highlighted by circles in the free energy profiles. The large standard deviation of the per-residue free energy change is caused by the structural asymmetry of homologous subunits.

The bovine glutamate dehydrogenase (BGDH) and the glucosamine-6-phosphate deaminase (G6PD) from *E*.*coli*, are large homohexamers consisting of a dimer of trimers, and a trimer of dimers, respectively. The well-studied allosteric regulation of BGDH features a repertoire of metabolites as well as several characterized allosteric sites [39, 40]. It is known that binding of glutamate (GLU) substrate and NADP+ (NDP) cofactor to the catalytic cleft elicits large-scale conformational changes in this complex molecular machine. For example, the antenna region (Fig 5A), which consists of intertwined alpha helices and is evolutionarily conserved in the animal kingdom, is essential to the allostery of BGDH. The antenna serves as the inter-subunit relay of allostery, and its motion is regulated by the allosteric activator ADP and inhibitor GTP. Remarkably, we observed the large increase in the free energy of allosteric response (5 kcal/mol) in the antenna region upon perturbation of the catalytic sites, in comparison to the small background free energy change ($\Delta g_{BGDH}^{\left(6\times GLU/NDP\right)}$ = 0.31 kcal/mol, Fig 5A and Table 1). On the other hand, a weak response is detected in the ADP and GTP binding sites ($\Delta g_{ADP}^{\left(6\times GLU/NDP\right)}$ = 0.18 kcal/mol and $\Delta g_{GTP}^{\left(6\times GLU/NDP\right)}$ = -0.18 kcal/mol, Table 1). The configurational work exerted in the antenna region is consistent with experimental data, which shows rotation of the helices as the bound catalytic cleft closes [39, 40]. We found that, in agreement with experimental data in which the presence of antenna region was shown to be required for allosteric signalling, this region plays a role of the allosteric modulator, suggesting, in turn, the presence of latent allosteric sites in this region (Fig 5A).

In the case of homohexameric G6PD (Fig 5B), the allosteric response results in the positive free energy change globally distributed in the complex upon perturbation in all catalytic sites (alpha-D-glucosamine 6-phosphate (AGP), $\Delta g_{G6PD}^{\left(6\times AGP\right)}$ = 0.66 kcal/mol), including the inter-subunit interface where the allosteric activator N-acetylglucosamine 6-phosphate (16G) binds ($\Delta g_{16G}^{\left(6\times AGP\right)}$ = 0.78 kcal/mol, Table 1). In particular, residues at the core appeared to be highly affected by the perturbation, especially Cys219, which forms disulphide bridges between subunits. Perturbing the catalytic site of one subunit essentially freezes the entire subunit including the proximal allosteric site for the activator 16G, increasing the free energy in the remaining unperturbed subunits (S1 Fig). It was shown in X-ray crystallography [41] and fluorescence spectroscopy [42] experiments that the substrate binding to the catalytic site of G6PD induces structural and dynamic changes in all subunits of the protein, consistent with our observations.

The classical set of proteins analysed here contains an equal proportion of protein structures in the apo and bound forms. Using crystal structures of PFK and PGDH with or without bound ligands, we show that the free energy profiles are largely similar (S2 and S3 Figs), allowing to work with only one available structure. In all proteins of the classical set, a positive free energy difference was detected in distant allosteric sites upon perturbation of the substrate and/or cofactor binding, as well as an anticipated negative one in the regions where the perturbation is applied. Despite the different modes of allosteric regulation, binding sites for both allosteric activator and inhibitor exhibit an increase of configurational work exerted upon perturbation of the functional ones. For example, in enzymes with overlapping binding sites for activator and inhibitor, such as the ATCase (Fig 2B) and the PFK (Fig 3C), both sites show positive Δ*g*_*i*_ values. Therefore, the positive free energy difference as a result of the functional site perturbation serves in our approach as the standard quantitative indicator for the allosteric sites.

The profiles of the free energy changes due to the reverse allosteric signalling upon perturbation at the functional sites typically show rather extensive areas of the positive free energy difference, which encompass the binding sites of known allosteric effectors. Therefore, the locations that yield a large free energy change can, actually, be allosterically coupled to corresponding catalytic site, and, therefore, can potentially contain unknown/latent allosteric sites. For example, the reverse perturbation analysis suggests the presence of latent allosteric sites in the large TrpE subunit of AnthS (Fig 2A), in the cAMP-binding domain of CAP (Fig 2C), in PTP1B (Fig 4A), as well as in the dimerization interface in NADME (Fig 3B), PFK (Fig 3C) and ThrS (Fig 4C). Furthermore, the antenna region in BGDH (Fig 5A) and multiple locations in G6PD (Fig 5B) can also contain latent allosteric sites that provide complex allosteric regulation of these large protein complexes.

### Operational definition of an allosteric site in the framework of the elastic network model

Fig 5 shows two cases, BGDH and G6PD, in which it was not possible to identify known regulatory sites proximal to the perturbed catalytic ones. In these proteins, the perturbation of the catalytic site stabilizes the region proximal to the binding sites due to the direct interaction of residues in these adjacent sites. These cases show that the separation of allosteric and the regulated functional sites is instrumental in allosteric communication, as postulated in the seminal Monod-Changeux-Jacob paper [43] that allosteric proteins should “…possess two, or at least two, stereospecifically different, non-overlapping receptor sites.” Therefore, for any high-throughput analysis and prediction of regulatory exosites, it is crucial to introduce a quantitative measure for spatial separation between catalytic and allosteric sites.

**Fig 6:**
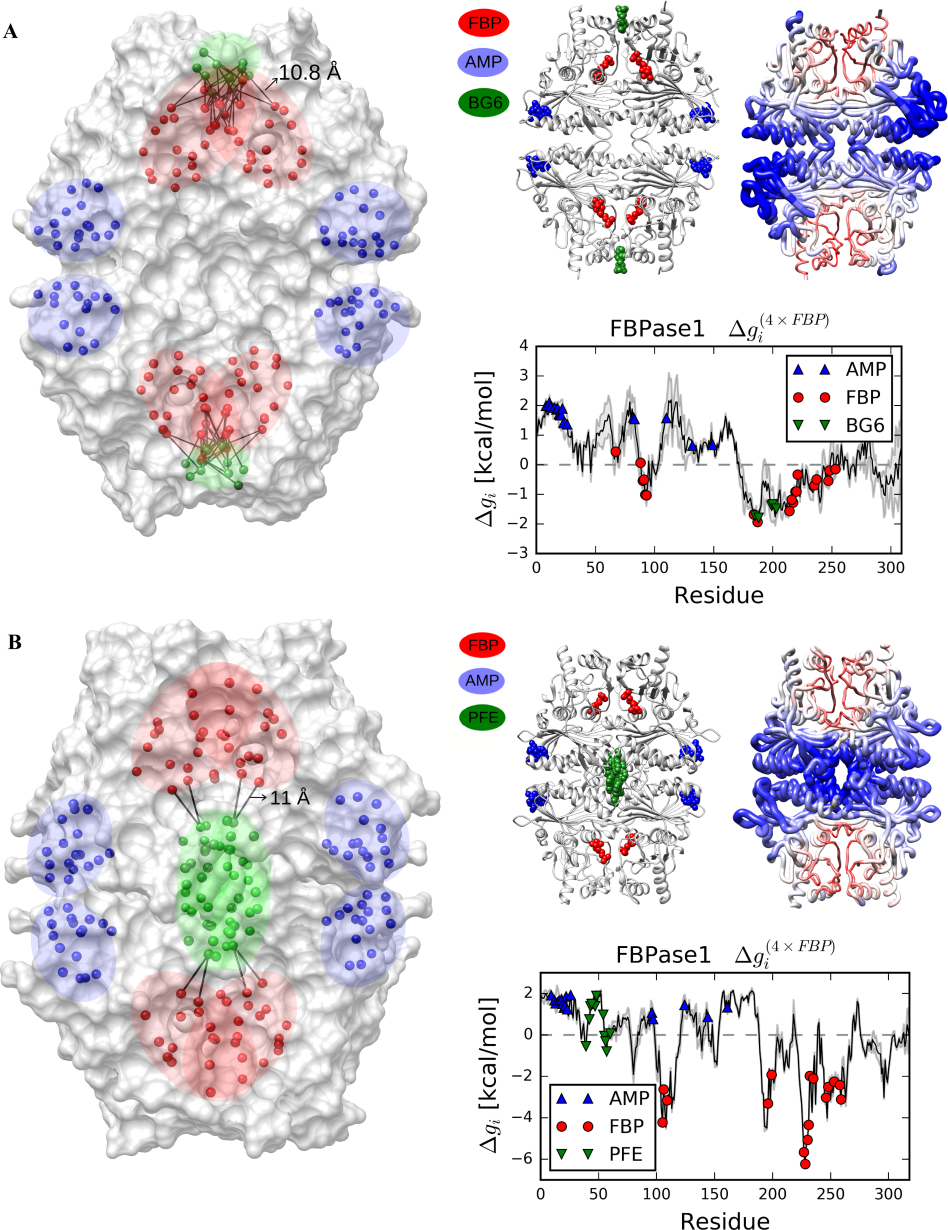
The operational definition of allosteric sites. The homologous FBPase1 from (A) *E*. *coli* and (B) *Sus scrofa* are used for the illustration of the communication versus physical interactions between residues of functional and allosteric sites. Residues in ligand binding sites are indicated by spheres on the left. Pairs of residues in different sites, with C_α_ pairwise distance not greater than 11 Å are shown by black lines. On the right, ligands from the ligand-bound crystal structures are superimposed to the analysed apo structures to show the location of the binding sites. Free energy changes of every residue upon reverse perturbation are calculated and displayed on the structures (colors and tube thickness) and in the free energy profiles.

Here, we have devised an operational definition of the allosteric site in the framework of the elastic network model of protein. To obtain a quantitative criterion for defining the allosteric site we introduce a notion of proximity, which is the fraction of interacting residues among all possible pairs that can be formed between residues of two sites–functional and the candidate allosteric ones. The distance cutoff for defining the interaction between residues is chosen according to the distance cutoff (11 Å) used in the microscopic allosteric potential of the original model [31] (see also Materials and Methods). Taking the most conservative approach, we have chosen the upper limit cutoff 11Å, which allows one to predict most of the allosteric sites. A site is considered allosteric if the corresponding proximity with the functional site does not exceed the threshold value (selection of the threshold value is explained below). We illustrate this definition by two homologous homotetramers, fructose 1,6-bisphosphatase 1 (FBPase 1) from *E*. *coli* (PDB ID: 2q8m, Fig 6A) and from *Sus scrofa* (PDB ID: 1kz8, Fig 6B), which control the gluconeogenesis pathway [44].

Adenosine monophosphate (AMP) and glucose-6-phosphate (BG6) bind to two distinct allosteric sites of FBPase 1 from *E*. *coli*, inhibiting hydrolysis of fructose 1,6-bisphosphate (FBP) [45]. The inhibitor binding at BG6 site would perturb the adjacent active FBP site via direct interaction between residues in these overlapping sites, whereas the distant AMP site regulates the FBP site by long-range allosteric signalling. Therefore, using the reverse perturbation approach, the distant AMP site can be readily identified based on the increase in free energy of allosteric response ($\Delta g_{AMP}^{\left(4\times FBP\right)}$ = 1.67 kcal/mol). However, simulated binding in the FBP site ($\Delta g_{FBP}^{\left(4\times FBP\right)}$ = -0.77 kcal/mol) decreases the free energy of the adjacent BG6 site ($\Delta g_{BG6}^{\left(4\times FBP\right)}$ = -1.62 kcal/mol), which is not a true allosteric site because of the 10% proximity to the FBP site (Fig 6A). Non-allosteric nature of interactions between the BG6 and FBP sites can be further confirmed by the weak decrease of the free energy observed upon direct perturbation via simulated binding to BG6 ($\Delta g_{FBP}^{\left(2\times BG6\right)}$ = -0.1 kcal/mol). On the contrary, the direct perturbation at the distant AMP (inhibitor) binding site increases the configurational work exerted at the FBP site ($\Delta g_{FBP}^{\left(4\times AMP\right)}$ = 0.44 kcal/mol), yielding the allosteric nature of communication between these sites.

The drug screening against FBPase 1 from *Sus scrofa* revealed that one of the anilinoquinazoline compounds, PFE, inhibits gluconeogenesis by binding to an allosteric site at the subunit interface [46]. Upon perturbing the catalytic FBP site, both AMP and PFE sites (with 0 and 2% proximity to the FBP site, respectively), displayed a similar increase in the free energy ($\Delta g_{AMP}^{\left(4\times FBP\right)}$ = 1.36 kcal/mol and $\Delta g_{PFE}^{\left(4\times FBP\right)}$ = 1.45 kcal/mol, Fig 6B). The configurational work exerted at the PFE site shows that the low proximity between functional and allosteric sites is a necessary condition for the correct definition of the latter. Additional analysis of several proteins that result in both successes and failures in detection of allosteric sites have led to the following operational definition: a site is considered allosterically coupled to the regulated catalytic one if the proximity between them is no more than 2%. The sites’ proximities obtained for each protein in the classical set (Table 1) show that all proteins have non-overlapping functional and allosteric sites except the BGDH and G6PD, where the proximities are 7 and 10%, respectively, hence failed to be detected as allosteric ones. This operational definition is important in order to correctly estimate the predictive power of reverse perturbation approach, allowing us to operate with proteins with different architectures and mutual locations of the functional and allosteric sites.

### Predictive power of the reverse perturbation analysis

**Fig 7:**
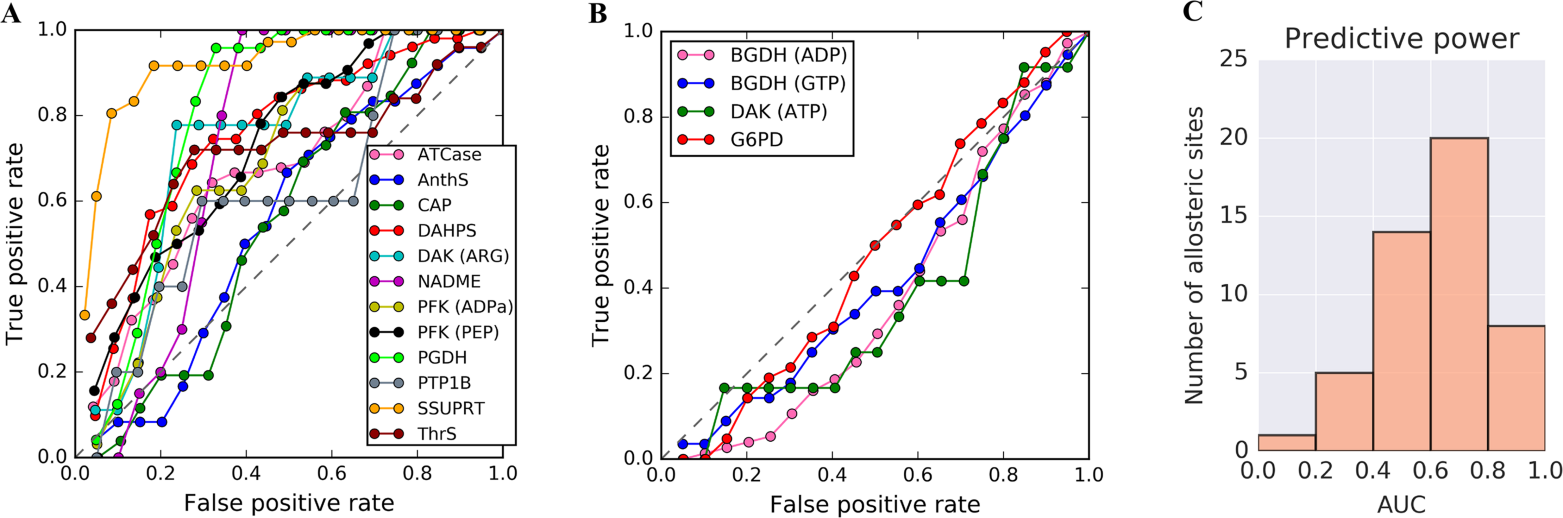
Receiver operating characteristic (ROC) curves. (A) High predictive power can be achieved for most allosteric proteins with distant functional and allosteric sites. (B) Allosteric sites for which low predictive powers are obtained. (C) A histogram of the area under the ROC curves (AUCs) for the detection of allosteric sites in proteins from the additional benchmark set of 41 proteins.

With the set of allosteric sites obtained on the basis of above operational definition, one can analyse the predictive power of the reverse perturbation approach. A successful prediction of allosteric sites is possible if their residues will exhibit a large free energy change upon perturbation of the functional site. The receiver operating characteristic (ROC) curve is used here to quantify the proportion of true positive (those that belong to a known allosteric site) and false positive (those not belonging to a known allosteric site) among residues with a large free energy change, upon perturbation at the corresponding functional sites (see Materials and Methods for details). Using the classical set of allosteric proteins, we show that the true positive rate increases more rapidly than the false positive rate for most allosteric sites, indicating that the majority of residues of known allosteric sites (true positives) are indeed located near the positive tail of the Δ*g*_*i*_ distribution (Fig 7A).

The known allosteric sites in the NADME (Fig 3B), PGDH (Fig 3D) and SSUPRT (Fig 4B) can be precisely determined based on the large increase in the free energy. It is important to emphasize, however, that presence of latent allosteric sites challenges the task of validating the prediction of the known ones, as some of the residues belonging to these sites can be erroneously scored as false positives. For example, the residues that show a large increase in Δ*g*_*i*_ values in potential allosteric sites, such as the antenna region in the BGDH, turn out to be false positives. Good predictive power can be achieved for known allosteric sites in the ATCase (Fig 2B), DAHPS (Fig 2D), DAK (Fig 3A), PFK (Fig 3C) and ThrS (Fig 4C). However, for the AnthS (Fig 2A), CAP (Fig 2C) and PTP1B (Fig 4A), the ROC curves are close to the diagonal line, indicating low predictive power for the known allosteric sites in these proteins (Fig 7A). This is likely due to the pronounced increase in free energy over the protein domains caused by catalytic site perturbation, suggesting the presence of latent allosteric sites that contribute to the false positives. For DAK (Fig 3A), the ROC curves for ARG (Fig 7A) and ATP binding sites in the free subunit (Fig 7B) vary greatly as the former site exhibits a higher increase in free energy, allowing its detection as the allosteric site. In the BGDH (Fig 5A) and G6PD (Fig 5B), the regulatory exosites are too close to the restrained catalytic site, hence, not satisfying the aforementioned operational definition (Fig 6B).

To complement the classical set of 13 allosteric proteins, we have used the operational definition of allosteric sites to obtain 41 proteins with 48 unique experimentally-determined allosteric sites from the benchmarking set of allosteric proteins [34] (S1 Table). We calculated the free energy profile for each of these proteins upon simulated ligand binding at the functional site (S4 Fig). Similar to the classical set, the predictive power of the reverse perturbation approach was estimated for each protein in this heterogeneous additional set. The area under the ROC curves (AUC) indicates good predictive power of the method (Fig 7C), showing that reverse perturbation approach allows one to successfully detect the majority of known allosteric sites in the additional set.

### Inducing and fine-tuning allosteric response

**Fig 8:**
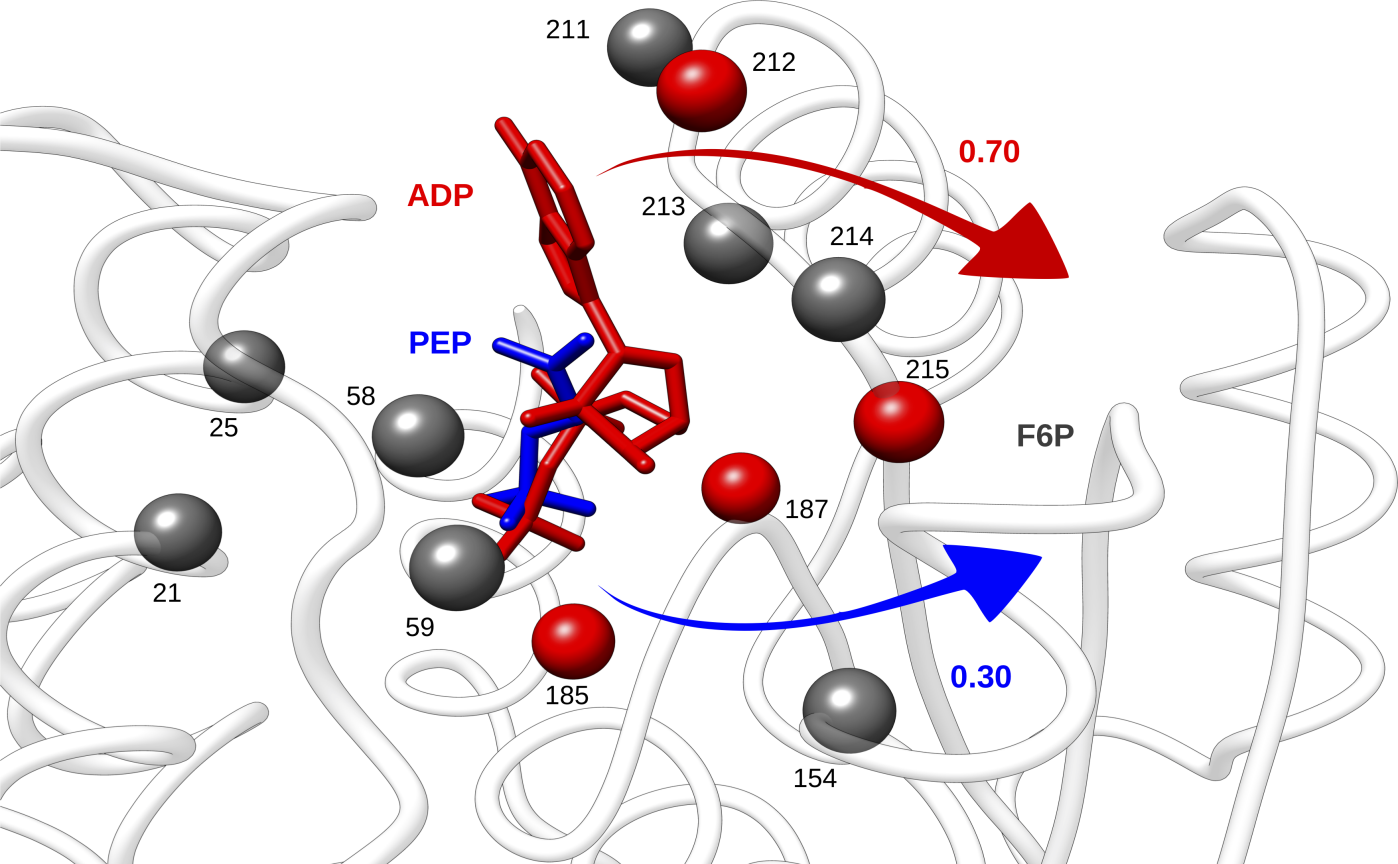
Allosteric signalling from overlapping activator/inhibitor binding sites in PFK. Perturbation of the larger binding site ADP (activator, red) causes larger increase in free energy (0.70 kcal/mol) to F6P functional sites, compared to that (0.30 kcal/mol) obtained from the smaller site PEP (inhibitor, blue). Residues in the sites are displayed as spheres along with the residue numbers. ADP and PEP from ligand-bound crystal structures (PDB code: 4pfk and 6pfk, respectively) are superimposed on the structure for illustration. The common residues that bind both ligands are colored grey, residues that only bind ADP are colored red. The distance cutoff for interacting residues of PFK and corresponding ligands is 3.5 Å.

In addition to the detection of known allosteric sites, the reverse perturbation approach delineates extended protein regions which are also characterized by the positive free energy change. This observation along with multiple indications of the presence of latent allosteric sites [5] in different proteins show that allosteric response can also be artificially induced by the interactions with rationally selected sets of residues belonging to latent or *de novo* designated allosteric sites. A classical allosteric enzyme, PFK (Fig 3C), which is regulated by the activator ADP and inhibitor PEP binding to the overlapping binding sites, serves as an excellent illustration that large difference in allosteric response can be caused by minor changes in the composition of an allosteric site (Fig 8). Specifically, ligand binding to the PEP site, which is a part of the larger ADP binding site results in a mild increase of free energy in the functional F6P site ($\Delta g_{F6P}^{\left(4\times PEP\right)}$ = 0.30 kcal/mol) compared to the global free energy change of the homotetramer ($\Delta g_{PFK}^{\left(4\times PEP\right)}$ = 0.10 kcal/mol). At the same time, a large increase in free energy is observed at F6P site upon simulated binding to ADP site ($\Delta g_{F6P}^{\left(4\times ADP\right)}$ = 0.70 kcal/mol, Fig 8). The modes of regulation in PFK, along with many other allosteric enzymes with overlapped activator and inhibitor binding sites in the literature [5] (see also, for example, ATCase, Fig 2B), highlights the importance of tuning allosteric response by varying the binding site composition.

We consider here as case studies, the subunit interfaces of the NADME (Fig 3B), ThrS (Fig 4C) and FBPase 1 (Fig 6A), which can apparently be used to induce a required allosteric response. In our model, an allosteric site consists of a set of residues whose perturbation results in the large free energy change in the corresponding functional site. In a given protein, to obtain a magnitude of regulation comparable with that of the native effectors, the allosteric response at the functional sites originating from the newly designated regulatory sites should be comparable with that obtained from known allosteric sites.

**Fig 9:**
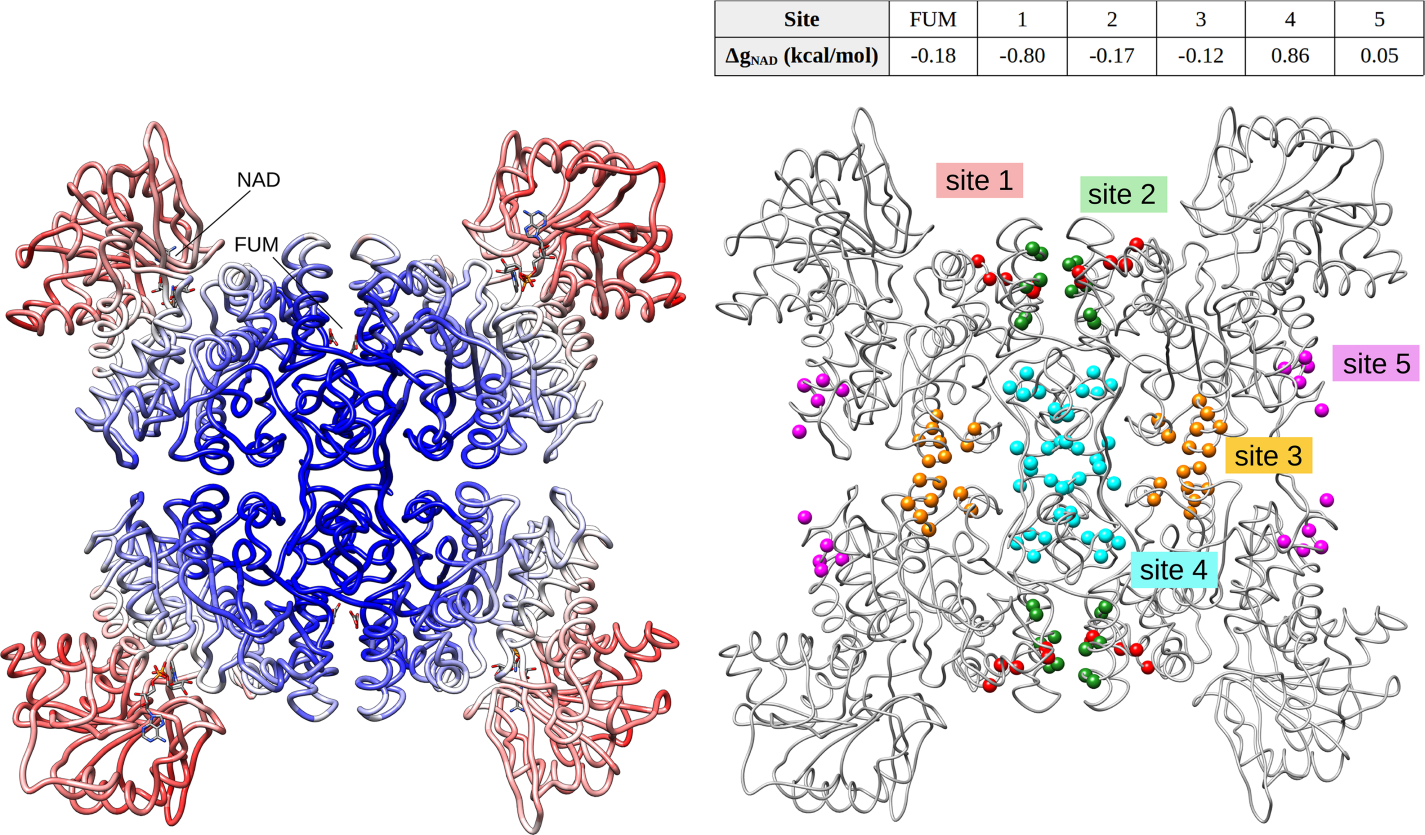
Inducing the allosteric response in the NADME. Left: reverse perturbation of allosteric communication in the NADME revealed presence of the repertoire of potential latent allosteric sites. Right: sites 1–5 are perturbed and the resulted free energy changes at functional NAD site are tabulated. Site 5 serves as a negative control.

The homotetrameric NADME is allosterically activated by the binding of fumarate (FUM) at the dimerization interface, causing a slight decrease in free energy at the catalytic site ($\Delta g_{NAD}^{\left(4\times FUM\right)}$ = -0.18 kcal/mol). Based on the reverse perturbation analysis in NADME (Fig 3B) and in order to illustrate the possibility to induce an allosteric response, four sites were putatively defined in the protein region corresponding to the increase of the free energy: site 1 (red) and site 2 (green) are located at the dimerization interface where fumarate binds, site 3 (yellow) is situated at the tetramerization interface where ATP binds to stabilize the functional tetrameric form of the enzyme [47], site 4 (cyan) is close to the tetramer’s core, and site 5 (magenta), which is located in the protein region with negligible free energy change is used as a negative control (Fig 9).

We show that a range of allosteric responses can be induced upon simulated binding to sites 1–4 for different modulation of the catalytic activity. Perturbation of site 1 induces a stronger decrease of the free energy in the catalytic site ($\Delta g_{NAD}^{\left(4\times site\;1\right)}$ = -0.80 kcal/mol) than that of the native FUM allosteric site (-0.18 kcal/mol). Similar to the effect of the FUM–site binding, perturbation of site 2 ($\Delta g_{NAD}^{\left(4\times site\;2\right)}$ = -0.17 kcal/mol) and the site 3 ($\Delta g_{NAD}^{\left(4\times site\;3\right)}$ = -0.12 kcal/mol) causes a slight decrease in the free energy in the catalytic site. On the other hand, simulated binding to site 4 strongly increases the free energy in the functional site ($\Delta g_{NAD}^{\left(4\times site\;4\right)}$ = 0.86 kcal/mol). The reverse perturbation analysis has also detected areas that are not allosterically coupled to the catalytic site, indeed simulated binding in site 5 (chosen as a negative control) induces only weak response in the catalytic site ($\Delta g_{NAD}^{\left(4\times site\;5\right)}$ = 0.05 kcal/mol). The generic nature of inducing allosteric response on the basis of the reverse perturbation approach is further illustrated with examples of allosteric signalling of different modes and magnitudes obtained in the cases of FBPase 1 (S5 Fig) and ThrS (S6 Fig).

**Fig 10:**
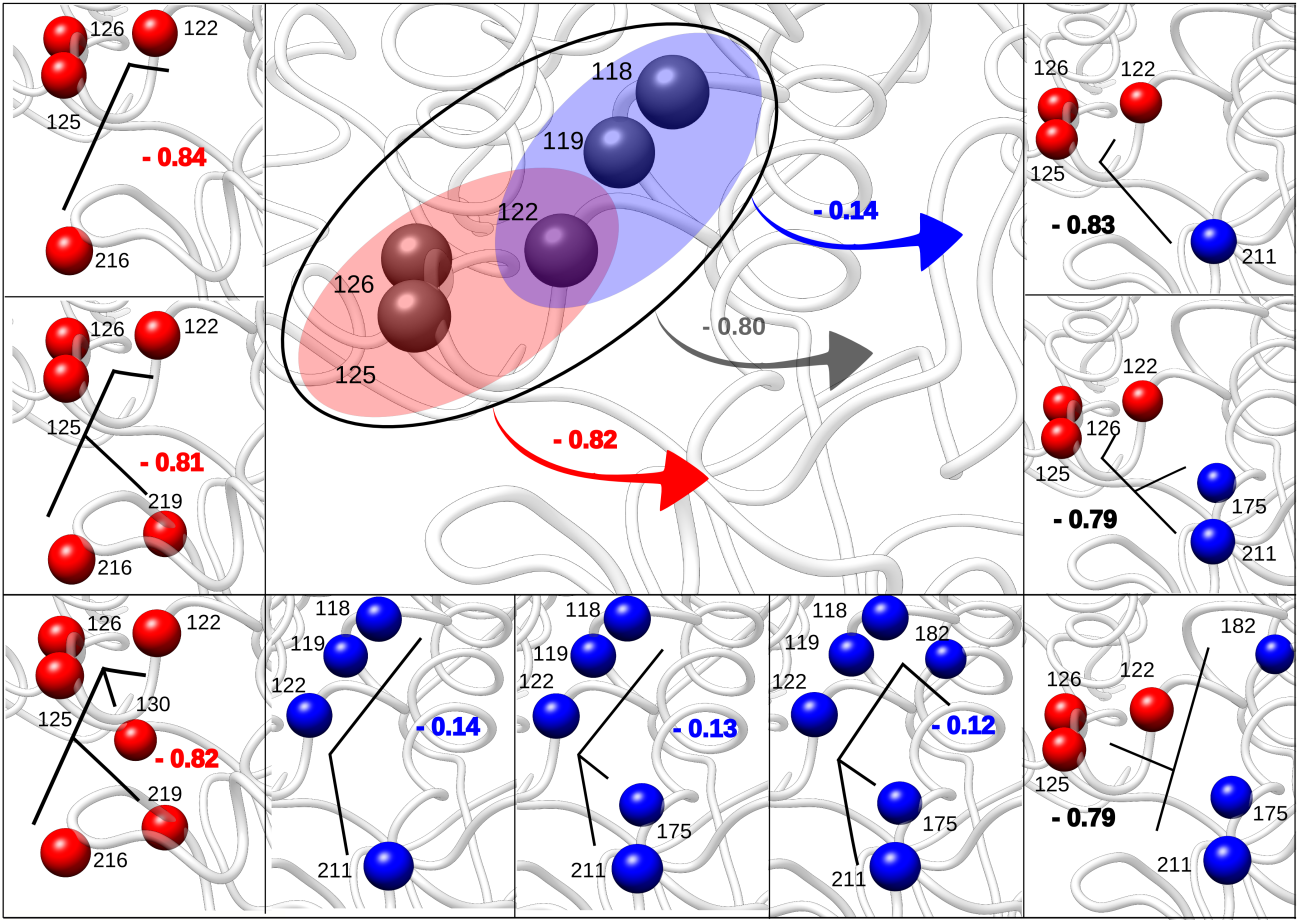
Fine-tuning of the allosteric response. Fine-tuning of allosteric response at the NAD functional site of the NAD-dependent malic enzyme can be achieved by varying composition of the perturbed site 1. The free energy changes (kcal/mol) at the NAD functional site are shown. The black lines indicate possible structures of ligand frameworks that can interact with corresponding allosteric sites.

Above analysis shows that in addition to the quest for latent allosteric sites, a more general question about the inducing of desired allosteric response can be formulated. Using site 1 of NAD-dependent malic enzyme as a test case, we show that the response can be further fine-tuned to achieve the necessary modulation in the catalytic site by varying the site’s composition (Fig 10).

Restricting perturbation of site 1 (blue) to a subset of residues (Leu118, Ala119, Gln122) substantially weakens the allosteric response (from -0.80 kcal/mol to -0.14 kcal/mol, Fig 10). Replacing Leu118 with the closest neighbours Gly117 and Ser121 recovers stronger allosteric signal (-0.67 kcal/mol and -1.06 kcal/mol, respectively, S7 Fig). Alternatively, considering the subset of residues Gln122, His125 and Ile126 (red) which produces an allosteric effect, which is comparable (-0.82 kcal/mol) to that induced by perturbing the whole site 1 (Fig 10). Allosteric signalling can also be fine-tuned to be weaker by replacing Gln122 with Ala119, Cys120 or Ser121, resulting in the allosteric responses -0.21 kcal/mol, -0.21 kcal/mol and -0.29 kcal/mol, respectively, at the regulated functional site (S7 Fig). Sites 1–5 are well separated from the distant NAD site with less than 2% proximity, which ensures the allosteric nature of signalling from these putatively designated sites.

Inducing and fine-tuning the allosteric response should be further complemented by the rational design or selection of allosteric effectors. For example, minimal sets of residues as the basis for targeted allosteric response (e.g. blue and red sets, Fig 10 (left, bottom) and S7 Fig) can be incrementally changed residue-by-residue (Fig 10) in order to achieve required strength of the allosteric signal. Alternatively, one can explore the repertoire of all possible binding sites, adjusting them for a given lead ligand (combined red-blue sets, Fig 10, right).

## Discussion

Allostery is a universal phenomenon where ligand-binding at a regulatory site causes a change in the ligand-affinity and/or activity at the coupled functional site [43]. Serious attempts to utilize the advantages of targeting allosteric sites instead of the orthosteric ones have only started in recent years, and the concept of allosteric drugs has since formed an important part in drug discovery [1, 2, 5, 6]. Prediction of allosteric sites that can remotely regulate the dynamics at the functional site of interest has been shown to be a challenging task [5, 15, 16]. In this paper we test the hypothesis of the reversibility of allosteric communication, according to which the perturbation at the functional site results in a signal that propagates towards allosterically active protein regions. We show that in most of the cases reverse perturbation at functional sites causes an increase of the free energy in protein regions that are dynamically coupled to them, which were subsequently used for the detection of allosteric sites. In general, the topology of the protein plays a non-trivial role in the propagation of the allosteric signal, especially when protein activity is regulated by more than one effectors. Using the protein set consisting of 13 classical allosteric proteins and an independent benchmark set of 41 proteins, we show that known allosteric sites can indeed be identified by the reverse perturbation method. Good predictive power of the method was obtained in all proteins of both classical and benchmark sets when the allosteric sites are spatially separated from the functional ones according to the operational definition of allosteric site, which, in turn, is based on the original Monod-Changeux-Jacob’s formulation [43] of allostery and relationship between the functional and the regulatory exosites.

After showing that known allosteric sites can be detected via the reverse perturbation method, we addressed the question of the allosteric sites identification. Using the NAD-dependent malic enzyme as an example, we show that simulated binding to two putatively defined sites in the protein region obtained from the reverse perturbation method (sites 2 and 3) results in the allosteric response at the functional site similar to that caused by the native effector fumarate. Moreover, the prediction of allosteric sites from the protein regions obtained via the reverse perturbation method may result in multiple solutions. A repertoire of overlapping and non-overlapping sites can induce comparable allosteric signals upon binding to these sites. This redundancy, suggests that the practical task on the detection of known and prediction of latent allosteric sites can be turned into the general problem of how to induce and fine-tune an allosteric response. The case studies of NAD-dependent malic enzyme, threonine synthase, and fructose-1,6-bisphosphatase were used here to show that a range of allosteric responses can be induced upon simulated binding in sites putatively defined in the regions with increased free energy obtained via the reverse perturbation approach. For example, while simulated binding in the fumarate and putatively designated sites 1–3 of NAD-dependent malic enzyme result in a free energy decrease at the catalytic site, perturbation in site 4 strongly increases the free energy in the catalytic site. Two additional examples, FBPase 1 and ThrS, further illustrate the possibility to adopt the reverse perturbation method for inducing the allosteric response. Further, a presence of the overlapping sites of activators and inhibitors, such as ADP and PEP in PFK, ATP and CTP in ATCase and many others, calls for the fine tuning of the induced allosteric signalling via the rational design of the interactions between ligand and binding site. We exemplify the fine-tuning of the allosteric effect by varying the composition of a designated binding site 1 of NAD-dependent malic enzyme, which results in different levels of the catalytic activity modulation. In general, knowing the allosteric response in the functional sites upon binding of the native allosteric ligand, it is possible to select new allosteric sites and/or ligands that cause the same effect as the natural ones and, therefore, can be considered as new regulatory exosites. Further, an exhaustive calculation of the allosteric effect caused by every residue would be instrumental for rationally defining the candidate/potential allosteric sites in the absence of preliminary experimental data. The latter can be obtained in mutation experiments that measure the allosteric effects of residue-by-residue substitutions on the protein activity [48], providing thus a foundation for the experimental verification and further improvement of the computational model.

Despite two major limitations of the current structure-based perturbation model, the lack of sequence information, and the coarse-grained description of proteins on the basis of structure-based C_α_ model, the reverse perturbation method should be regarded as a general strategy in finding and exploring allosteric sites. For example, a reverse perturbation-like strategy was used in a recent experimental work where hydrogen exchange mass spectroscopy method was used for characterizing the allosterically active regions of protein Hsp90 induced by the orthosteric binding [49]. It is important to note, that modularity of the structure-based perturbation model is instrumental for improving the accuracy of calculations, as it was done, for example, in Gehrig, S. *et al*. [50], where instead of using normal modes the slowest principal components (PCA) calculated from the MD trajectories were used for the calculations of the allosteric potential and the corresponding free energy derived in our model. However, increase of the accuracy by implementing the MD simulations will come at a price of the calculation speed. An alternative way of the model improvement would be via including the sequence dependence in the energy function, which preserves, at the same time, the model’s efficiency in terms of the high speed of calculations.

To conclude, the task of inducing and fine-tuning of allosteric response can be generalized and formulated in the following sequence of steps: (i) finding the potential regulatory exosites via reverse perturbation approach; (ii) optimizing the compositions/structures of the binding exosites that can induce a required allosteric signalling upon binding to them; (iii) selection of the appropriate ligands that interact with the chosen allosteric site with sufficient binding affinity; (iv) allocation of the regulatory exosites that provide required allosteric effect at the corresponding functional site in the case of pre-existing library of ligands. By exploring the possibility to detect known, finding latent, and designing new regulatory exosites and corresponding allosteric effectors, the reverse perturbation method introduced in this work provides a conceptual framework aimed at the optimization of the allosteric regulation of protein activity.

## Materials and methods

### Protein data set

We used here the set of 13 allosteric enzymes included in previous studies [31–33], which we refer to as the “classical set”. An additional set of 41 allosteric proteins with 48 experimentally-determined allosteric sites is obtained from the benchmarking collection of allosteric proteins ASBench [34]. In collecting above additional set, we applied the following requirements: (i) structures lacking information on functional sites, on parts of the structures, proteins that change the oligomerization state and structures in which regulation involves protein-protein interactions were omitted together with other cases of missing annotation; (ii) based on the operational definition of the allosteric site and applying the “proximity threshold” (see below) of no more than 2%, we obtained the final list of 48 sites in 41 proteins (S1 Table). Interacting residues were extracted from the structures, based on the distance cutoff of 4.5 Å between the heavy atoms of protein residues and those of the ligand. The quaternary structure assemblies were obtained from the PDBePISA [51, 52] with all water molecules, ions and ligands removed. The apo form of protein complexes was used whenever available, except for CAP due to the large structural difference between its apo and DNA-bound forms.

### Structure-based statistical mechanical model of allostery

The structure-based statistical mechanical model of allostery used in this work consists of three parts, which are described in detail in a previous work [31]. First, C_α_ harmonic models are built for the ligand-free (unperturbed) and ligand-bound (perturbed) systems from a single crystal structure. The presence of a bound ligand, i.e. perturbation, is modeled via the harmonic restrain of all residue pairs in the binding site. For the unperturbed system, the harmonic potential for all pairs of C_α_ atoms *i* and *j* is given by

$$E^{\left(0\right)}(r-r^{0})=\sum_{pairs\;i,j}k_{i,j}{\left(d_{i,j}-d_{i,j}^{0}\right)}^{2}$$ (1)

where ***r*** is the 3N-dimensional vector of coordinates of C_α_ atoms and ***r***^0^ is a vector of C_α_ atoms from the reference structure. The *d*_*i*,*j*_ is the distance between any pair of C_α_ atoms *i* and *j*, the corresponding distance in the reference structure is $d_{i,j}^{0}$. The distance-dependent force constant *k*_*i*,*j*_ decays as ${\left(1/d_{i,j}^{0}\right)}^{6}$ with a global cutoff of 25 Å [53]. The potential associated with the ligand-bound state (B) with *n* bound sites *S* = {*s*_1_,*s*_2_,…,*s*_*n*_} is given by

$$E^{\left(B\right)}\;(r-r^{0})=E^{\left(0\right)}\;(r-r^{0})+\alpha \sum_{n}\sum_{pairs\;i,j\in s_{n}}k_{i,j}{\left(d_{i,j}-d_{i,j}^{0}\right)}^{2}$$ (2)

where *α* = 100 is a stiffening factor of the perturbed site. Protein’s configurational ensembles are characterized by the first 10 slowest normal modes $e_{\mu }^{\left(0\right)}$ and $e_{\mu }^{\left(B\right)}$ for the free and ligand-bound systems, respectively. Previous studies have shown that the conformational transitions in allosteric communication are well described by the first 10 low frequency normal modes [32, 33].

Second, we define a microscopic allosteric potential associated with a residue *i*

$$U_{i}(\sigma )=\frac{1}{2}\sum_{\mu }\varepsilon_{\mu ,i}\sigma_{\mu }^{2},$$ (3)

which measures the total elastic work acting on a residue *i* as a result of the change in displacements of all its neighbours caused by the normal mode *μ*. The generic configuration of residue *i* is obtained from the reference configuration $r_{i}^{0}$ by superimposing the vectors ***e***_*μ*,*i*_ such as $r_{i}(\sigma )=r_{i}^{0}+\sum_{\mu }\sigma_{\mu }e_{\mu ,i}$, where *σ* = (*σ*_1_,…,*σ*_*μ*_,…) is a vector of Gaussian amplitudes. Thus, the residue configuration ***r***_*i*_(*σ*) is uniquely identified by the state vector (*σ*_1_,…,*σ*_*μ*_,…). The parameters *ε*_*μ*,*i*_ measures the elastic stress on the residue *i* and its neighbours *j* as result of the motion associated with the mode ***e***_*μ*_

$$\varepsilon_{\mu ,i}=\sum_{j:d_{i,j}^{0}<d_{c}}c{\left|e_{\mu ,i}-e_{\mu ,j}\right|}^{2},$$ (4)

where *c* = 1 kcal/mol/Å^2^ and a distance cutoff *d*_*c*_ = 11 Å. The allosteric potential in Eq 3 is evaluated for both protein states, ligand free (0) and ligand bound (B) ones, respectively. Finally, in order to obtain a per-residue free energy, the allosteric potential is integrated over all possible configurations *σ*, resulting in the partition function $z_{i}=\Pi_{\mu }{\left(\frac{2\pi k_{B}T}{\varepsilon_{\mu ,i}}\right)}^{1/2}$, and, consecutively in the free energy *g*_*i*_ = −*k*_*B*_*T* ln *z*_*i*_. The per-residue free energy difference between the unperturbed (0) and perturbed (B) protein states is

$$\Delta g_{i}=\frac{1}{2}k_{B}T\sum_{\mu }\ln\frac{\varepsilon_{\mu ,i}^{\left(B\right)}}{\varepsilon_{\mu ,i}^{\left(0\right)}}.$$ (5)

To compare the relative strength of the free energy change *Δg*_*i*_ for one residue to the effects on the corresponding subunit, the following average is considered

$$\Delta g_{U}=\frac{1}{n_{U}}\sum_{i\in U}\Delta g_{i},$$ (6)

where *n*_*U*_ is the total number of residues in the subunit *U*.

### Reverse perturbation analysis

In the reverse perturbation approach, the functional sites are perturbed, and, as a result, the change in the free energy at the levels of residues and sites is evaluated throughout the protein. To analyse the Δ*g*_*i*_ values of every residue in the oligomeric enzyme and to obtain the corresponding Δ*g*_*i*_ profile, the Δ*g*_*i*_ of corresponding residues from different subunits are averaged. Both functional and allosteric sites are indicated on the Δ*g*_*i*_ profile (Figs 2–6 and S1–S4 Figs). The change of the free energy in the site *s* is also estimated as the average of the free energy changes among the residues in this site

$$\Delta g_{S}=\frac{1}{n_{s}}\sum_{i\in s}\Delta g_{i},$$ (7)

where *n*_*s*_ is the total number of residues in the site *s*. Eq 7 is used to obtain the free energy changes for every functional and allosteric site.

### Operational definition of the allosteric site

The operational definition of the allosteric site is based on the restriction on a spatial proximity of communicating functional and regulatory sites. For every pair of residues *i* and *j*, the number of physically interacting pairs on the basis of the distance cutoff *d*_*c*_ = 11 Å is obtained. The proximity is defined as the fraction of interacting pairs over the total number of pairs between the residues of functional and candidate allosteric sites under consideration:

$$PX=\frac{n_{d_{i,j}<d_{c}}}{n_{i,j}}\times 100\%$$ (8)

A ligand-binding site is defined as allosteric for the corresponding functional site within the same subunit if the proximity *PX* is no more than 2%.

### Receiver operating characteristic curve (ROC) analysis

A distribution of the free energy changes Δ*g*_*i*_ is obtained upon perturbation of the catalytic sites for each protein in the classical and benchmark sets. For most of the allosteric proteins, all residues in the known allosteric sites exhibit an increase in the free energy, hence only the positive range of the Δ*g*_*i*_ distribution is used. In the PTP1B and BGDH, the entire Δ*g*_*i*_ distributions are used as the known allosteric sites contain equal proportion of residues with gain or loss of the free energy. For plotting the ROC, the first bin corresponding to the residues within the top 5% of the Δ*g*_*i*_ distribution is first used. A sequence of bins with decreasing thresholds with a 5% step is defined to obtain a series of true and false positive rates of the ROC curve. For residues with Δ*g*_*i*_ above the threshold, a true positive is scored if the residue in the crystal structure is located within 4.5 Å from the allosteric effector, whereas a false positive indicates that the residue does not belong to a known allosteric site.

### Computational framework and illustration

The harmonic models of proteins and the normal modes analysis are performed using the MMTK package [54]. Fourier approximation is used in the calculation of normal modes [55]. UCSF Chimera [56] is used to generate the illustrations.

## Supporting information

S1 Fig Reverse perturbation of a single functional site in glucosamine-6-phosphate deaminase. Restraining the functional AGP site in glucosamine-6-phosphate deaminase (G6PD) stabilizes the entire protein subunit.
(TIFF)

S2 Fig Free energy profiles for different apo and ligand-bound structures of PFK. Free energy profiles are calculated for the apo structure of PFK (PDB: 3pfk), the PFK structure with activator ADP and F6P bound (PDB: 4pfk) and the bound form of PFK with an inhibitor PEP (PDB: 6pfk).
(TIFF)

S3 Fig Free energy profiles for different apo and ligand-bound structures of PGDH. For PGDH, AKG and NAD bound structure (PDB: 1yba) and SER bound structure (PDB: 1psd) are used.
(TIFF)

S4 Fig Free energy profiles for 41 allosteric proteins from the benchmark set. Residues of the explored functional and allosteric sites are marked by different shapes and colors.
(PDF)

S5 Fig Inducing the allosteric response in fructose 1,6-phosphatase 1. Reverse perturbation of allosteric communication reveals a repertoire of potential latent allosteric sites in the subunit interfaces of fructose 1,6-phosphatase (FBPase 1). Sites 1–3 in FBPase 1 are perturbed, and the resulted free energy changes at functional FBP site are tabulated. Site 4 serves as a negative control.
(TIFF)

S6 Fig Inducing the allosteric response in threonine synthase. Reverse perturbation of allosteric communication reveals a repertoire of potential latent allosteric sites in the subunit interfaces of threonine synthase (ThrS). Sites 1–3 in ThrS are perturbed, and the resulted free energy changes at functional PLP sites are tabulated. Site 4 serves as a negative control.
(TIFF)

S7 Fig Fine-tuning of the allosteric response in NAD-dependent malic enzyme. The compositions of red and blue subsites in the site 1 of NAD-dependent malic enzyme (NADME) are varied on a residue-by-residue basis for the fine-tuning of allosteric response exerted at the functional NAD site.
(TIFF)

S1 Table A list of 41 allosteric proteins with 48 allosteric sites obtained from the ASBench database [34]. The list is obtained on the basis of the operational definition of allosteric sites. Predictive power for known allosteric sites in the protein, which is quantified by the area under the ROC curves (AUCs) for positive free energies, is given in the last column.
(DOCX)
