# Supplementary figures and images for: Reversing allosteric communication: From detecting allosteric sites to inducing and tuning targeted allosteric response
Source: PLoS Comput Biol. 2018 Jun 18;14(6):e1006228. doi: 10.1371/journal.pcbi.1006228 (PMC6023240; doi:10.1371/journal.pcbi.1006228)

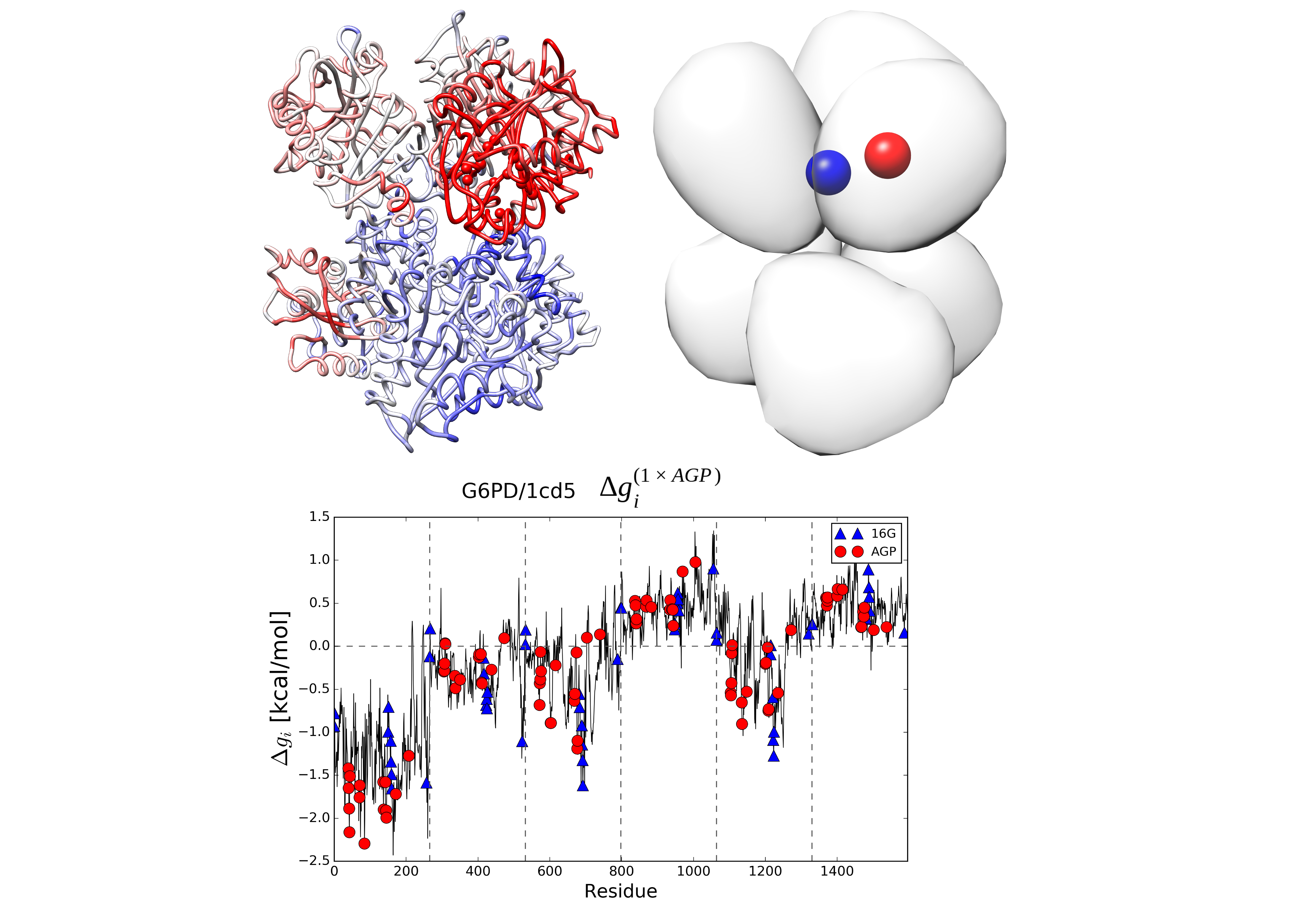

Supplement: S1 Fig — Restraining the functional AGP site in glucosamine-6-phosphate deaminase (G6PD) stabilizes the entire protein subunit. (TIFF) [file pcbi.1006228.s001.tiff]

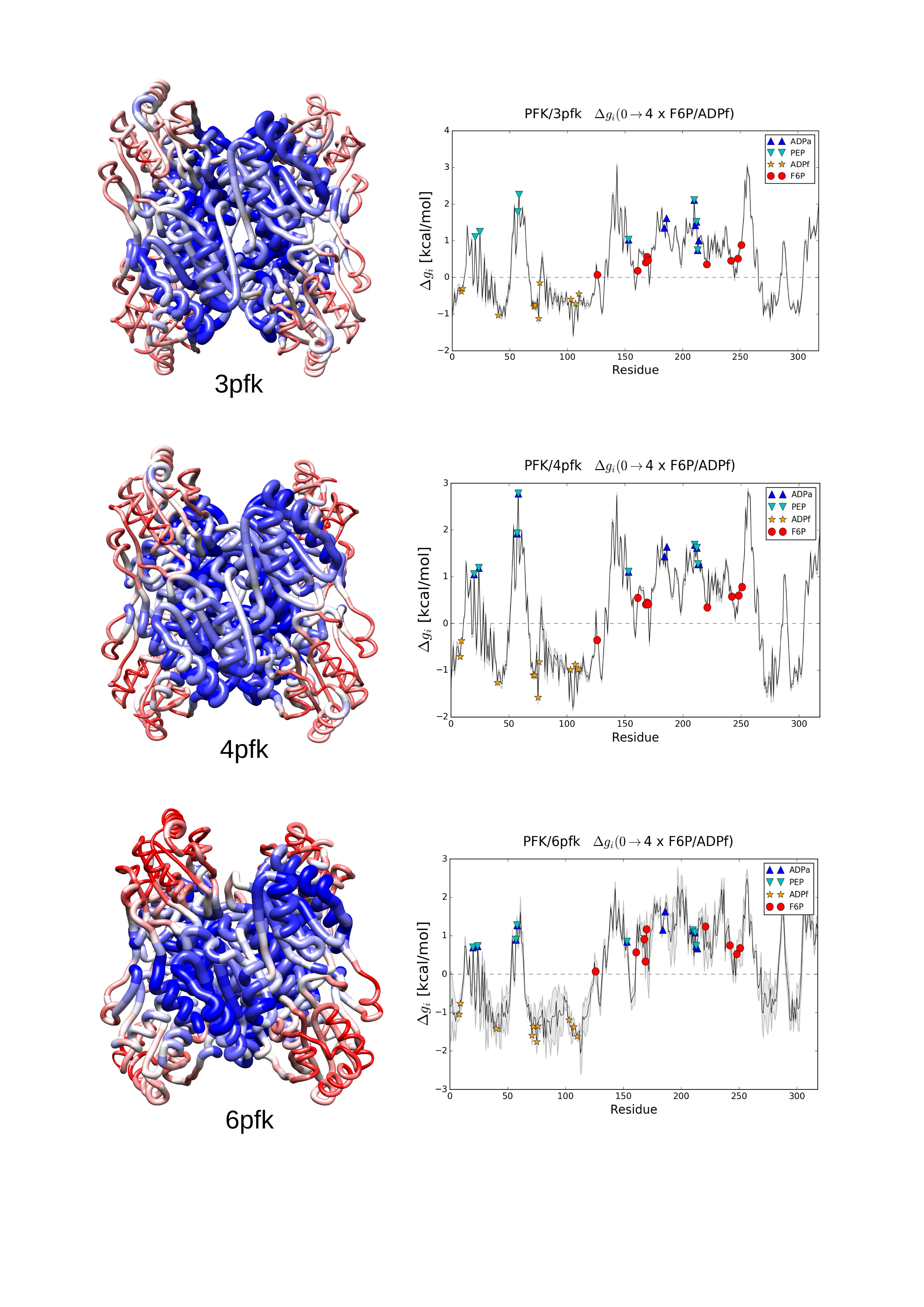

Supplement: S2 Fig — Free energy profiles are calculated for the apo structure of PFK (PDB: 3pfk), the PFK structure with activator ADP and F6P bound (PDB: 4pfk) and the bound form of PFK with an inhibitor PEP (PDB: 6pfk). (TIFF) [file pcbi.1006228.s002.tiff]

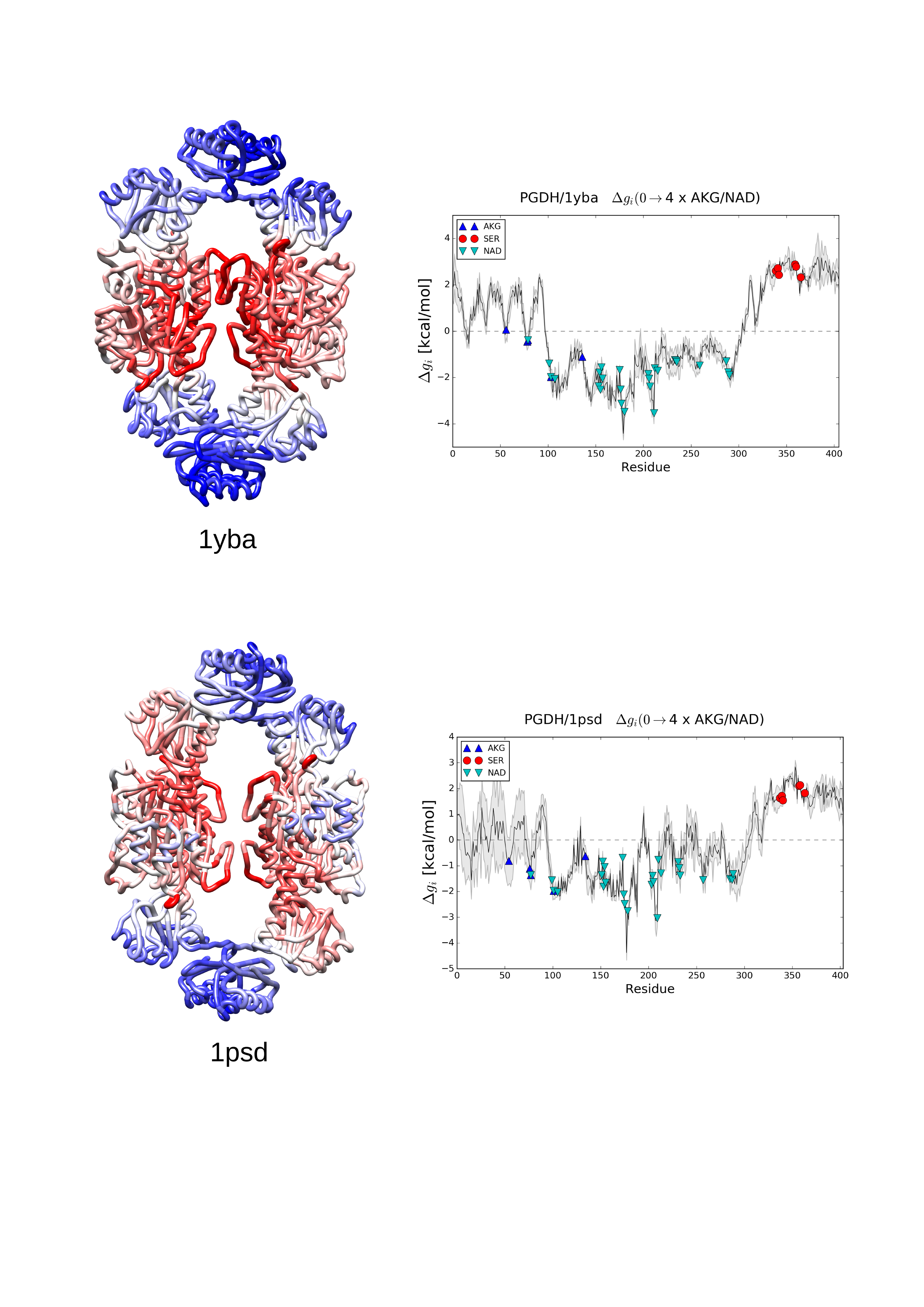

Supplement: S3 Fig — For PGDH, AKG and NAD bound structure (PDB: 1yba) and SER bound structure (PDB: 1psd) are used. (TIFF) [file pcbi.1006228.s003.tiff]

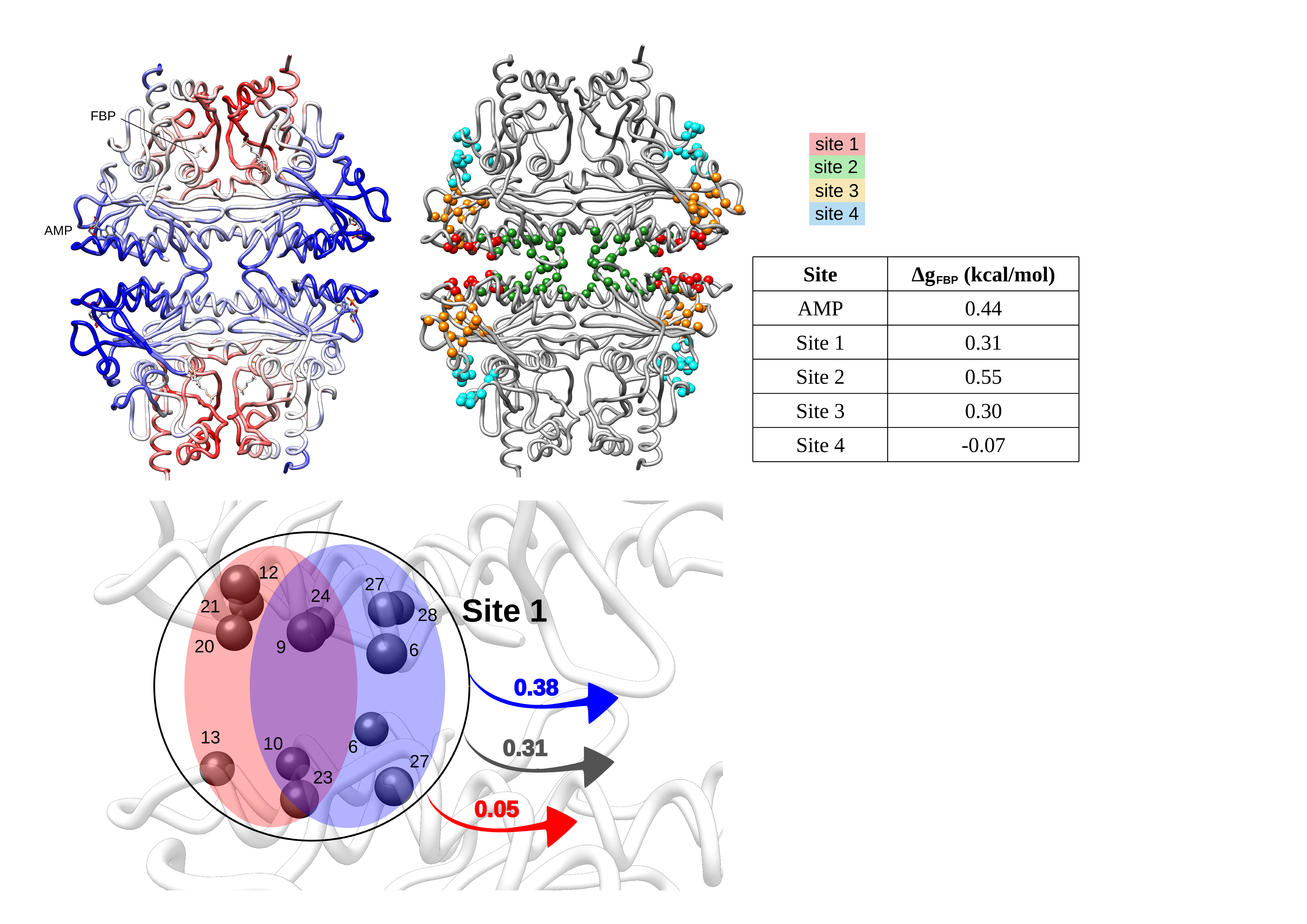

Supplement: S5 Fig — Reverse perturbation of allosteric communication reveals a repertoire of potential latent allosteric sites in the subunit interfaces of fructose 1,6-phosphatase (FBPase 1). Sites 1–3 in FBPase 1 are perturbed, and the resulted free energy changes at functional FBP site are tabulated. Site 4 serves as a negative control. (TIFF) [file pcbi.1006228.s005.tiff]

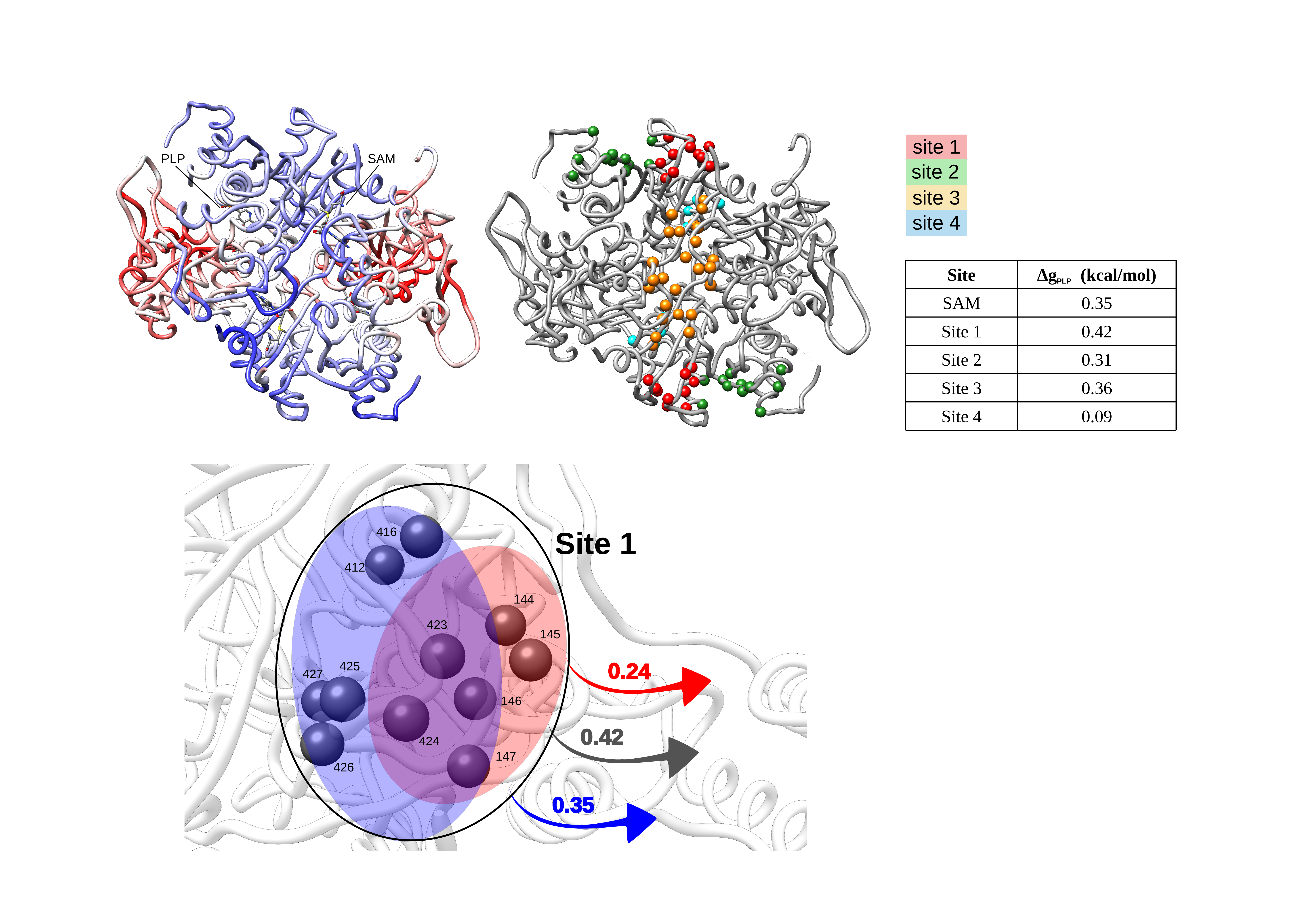

Supplement: S6 Fig — Reverse perturbation of allosteric communication reveals a repertoire of potential latent allosteric sites in the subunit interfaces of threonine synthase (ThrS). Sites 1–3 in ThrS are perturbed, and the resulted free energy changes at functional PLP sites are tabulated. Site 4 serves as a negative control. (TIFF) [file pcbi.1006228.s006.tiff]

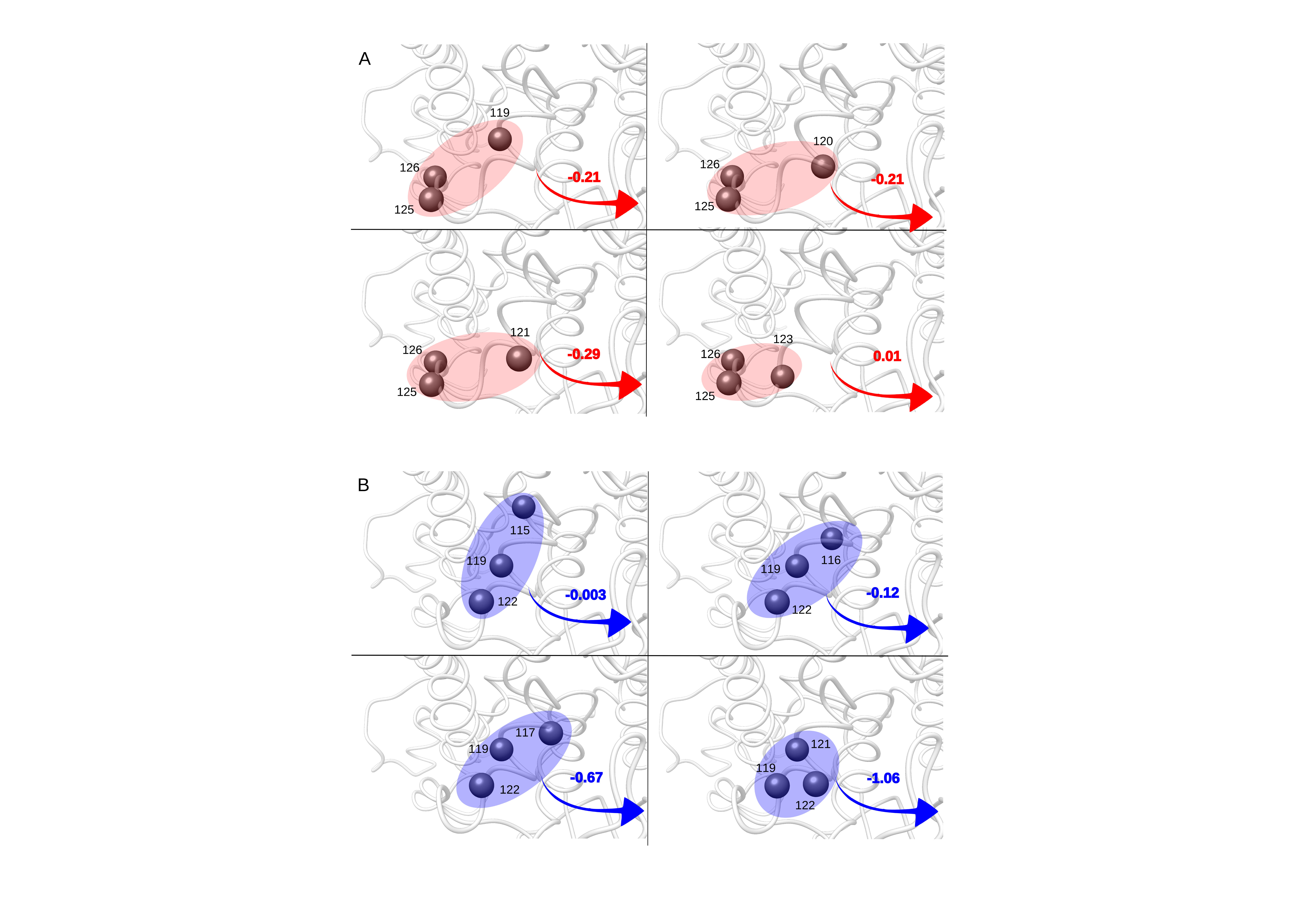

Supplement: S7 Fig — The compositions of red and blue subsites in the site 1 of NAD-dependent malic enzyme (NADME) are varied on a residue-by-residue basis for the fine-tuning of allosteric response exerted at the functional NAD site. (TIFF) [file pcbi.1006228.s007.tiff]
