# Supplementary material for: Reversing allosteric communication: From detecting allosteric sites to inducing and tuning targeted allosteric response
Source: PLoS Comput Biol. 2018 Jun 18;14(6):e1006228. doi: 10.1371/journal.pcbi.1006228 (PMC6023240; doi:10.1371/journal.pcbi.1006228)
Supplement: S4 Fig — Residues of the explored functional and allosteric sites are marked by different shapes and colors. (PDF) [file pcbi.1006228.s004.pdf]

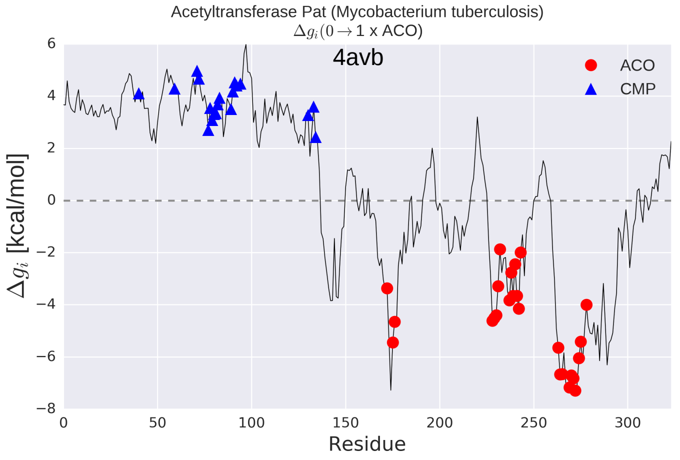
Acetyltransferase Pat (Mycobacterium tuberculosis)
$\Delta g_i(0 \rightarrow 1$ x ACO)
4avb
ACO
CMP
Δg_i [kcal/mol]
Residue

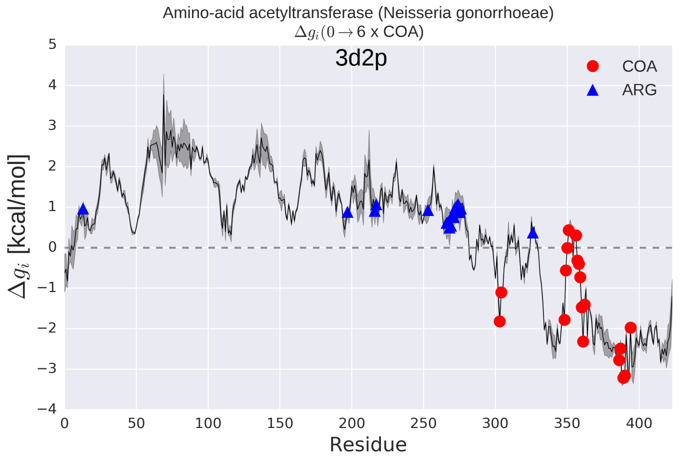
Amino-acid acetyltransferase (Neisseria gonorrhoeae)
$\Delta g_i(0 \rightarrow 6$ x COA)
3d2p
COA
ARG
Δg_i [kcal/mol]
Residue

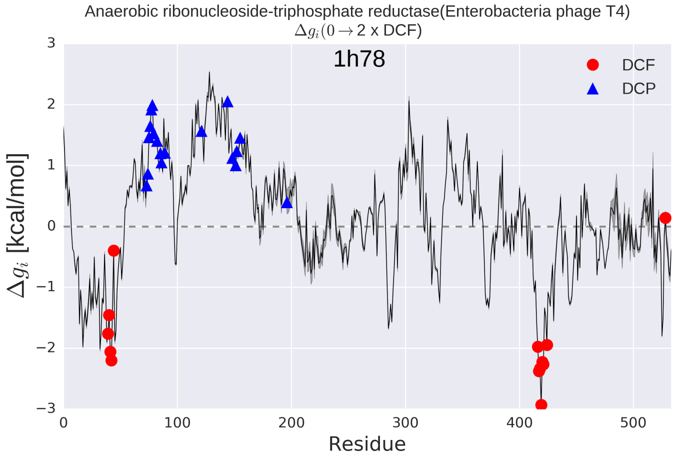
Anaerobic ribonucleoside-triphosphate reductase(Enterobacteria phage T4)
$\Delta g_i(0 \rightarrow 2$ x DCF)
1h78
DCF
DCP
Δg_i [kcal/mol]
Residue

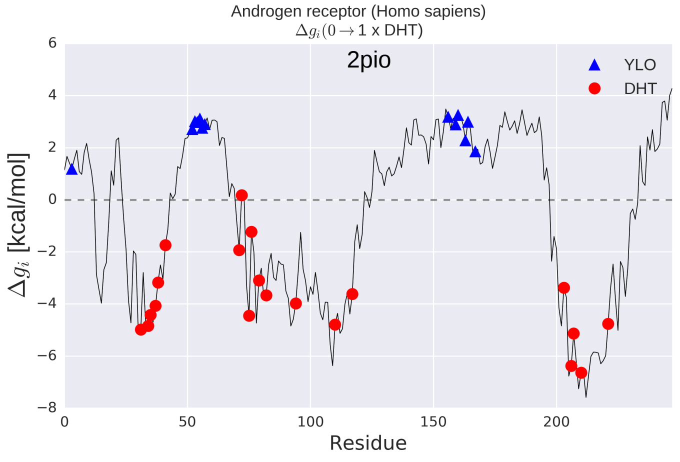
Androgen receptor (Homo sapiens)
$\Delta g_i(0 \rightarrow 1$ x DHT)
2pio
YLO
DHT
Δg_i [kcal/mol]
Residue

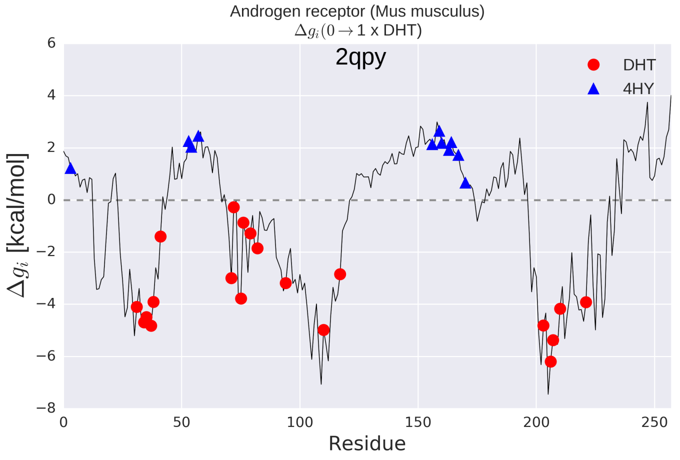
Androgen receptor (Mus musculus)
$\Delta g_i(0 \rightarrow 1$ x DHT)
2qpy
DHT
4HY
Δg_i [kcal/mol]
Residue

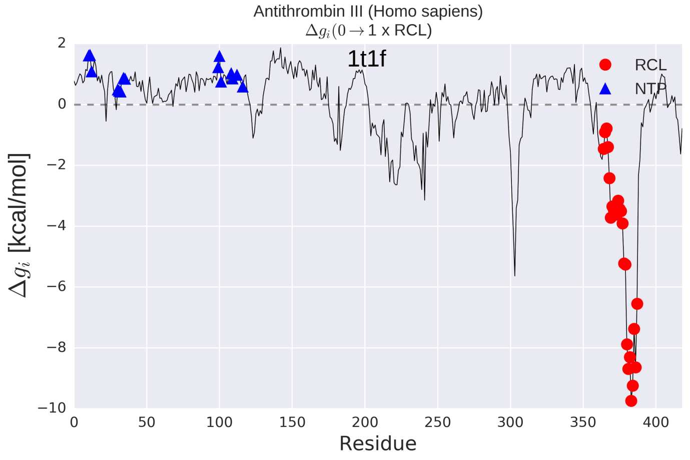
Antithrombin III (Homo sapiens)
$\Delta g_i(0 \rightarrow 1$ x RCL)
1t1f
RCL
NTP
Δg_i [kcal/mol]
Residue

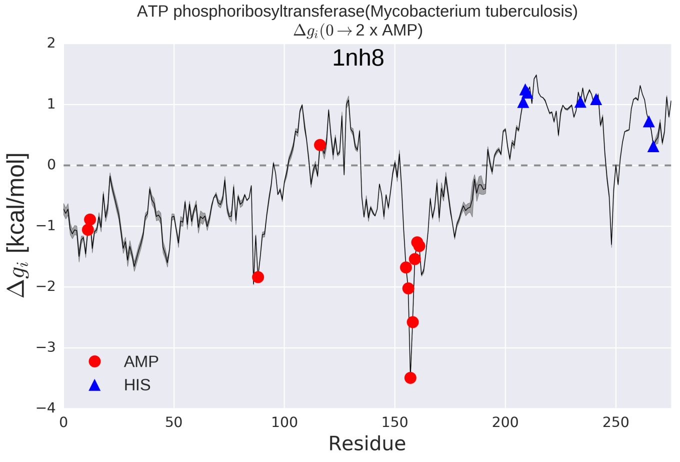
ATP phosphoribosyltransferase(Mycobacterium tuberculosis)
$\Delta g_i(0 \rightarrow 2$ x AMP)
1nh8
AMP
HIS
Δg_i [kcal/mol]
Residue

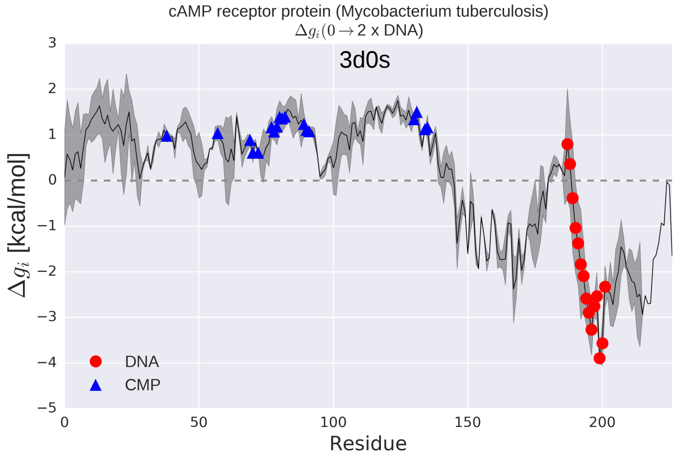
cAMP receptor protein (Mycobacterium tuberculosis)
$\Delta g_i(0 \rightarrow 2$ x DNA)
3d0s
DNA
CMP
Δg_i [kcal/mol]
Residue

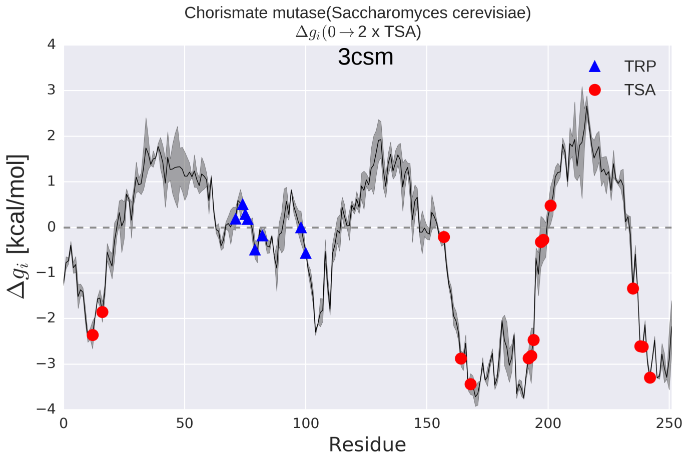
Chorismate mutase(Saccharomyces cerevisiae)
$\Delta g_i(0 \rightarrow 2$ x TSA$)$
3csm
TRP
TSA
Δg_i [kcal/mol]
Residue

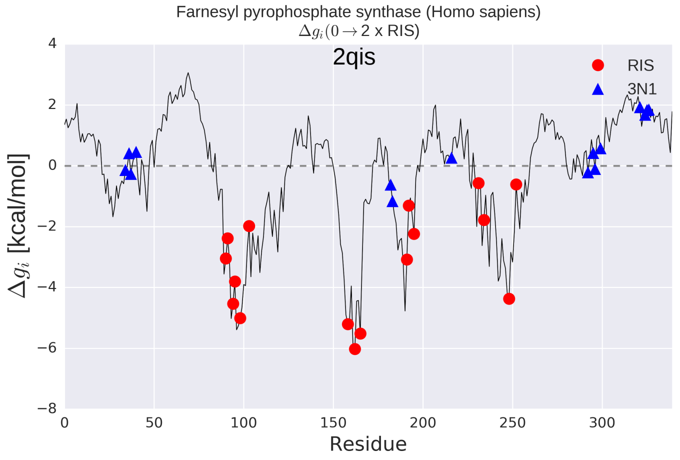
Farnesyl pyrophosphate synthase (Homo sapiens)
$\Delta g_i(0 \rightarrow 2$ x RIS$)$
2qis
RIS
3N1
Δg_i [kcal/mol]
Residue

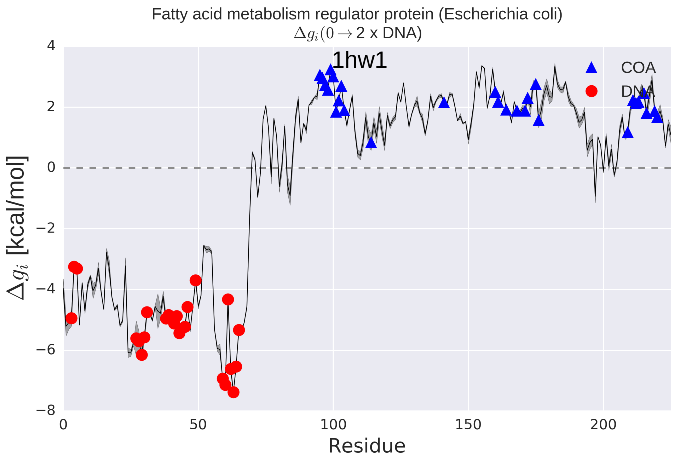
Fatty acid metabolism regulator protein (Escherichia coli)
$\Delta g_i(0 \rightarrow 2$ x DNA$)$
1hw1
COA
DNA
Δg_i [kcal/mol]
Residue

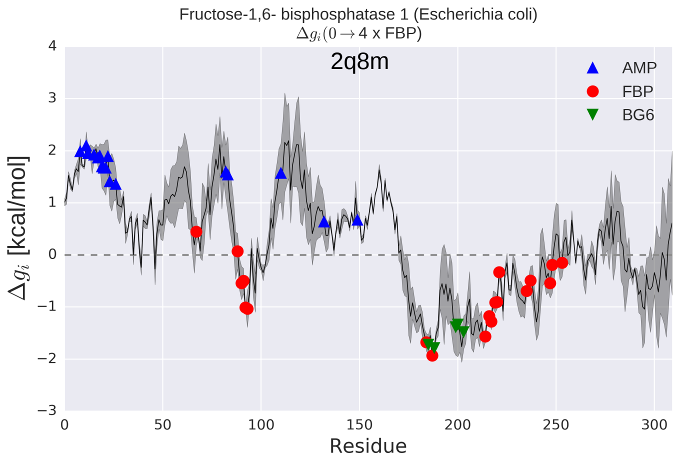
Fructose-1,6- bisphosphatase 1 (Escherichia coli)
$\Delta g_i(0 \rightarrow 4$ x FBP$)$
2q8m
AMP
FBP
BG6
Δg_i [kcal/mol]
Residue

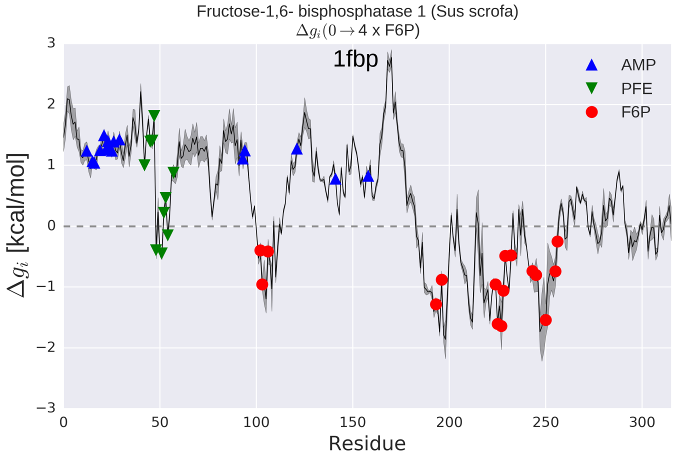
Fructose-1,6- bisphosphatase 1 (Sus scrofa)
$\Delta g_i(0 \rightarrow 4$ x F6P$)$
1fbp
AMP
PFE
F6P
Δg_i [kcal/mol]
Residue

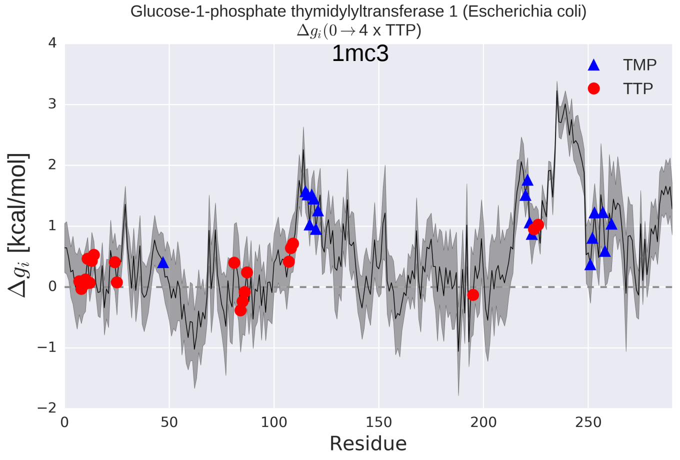
Glucose-1-phosphate thymidylyltransferase 1 (Escherichia coli)
$\Delta g_i(0 \rightarrow 4$ x TTP$)$
1mc3
TMP
TTP
Δg_i [kcal/mol]
Residue

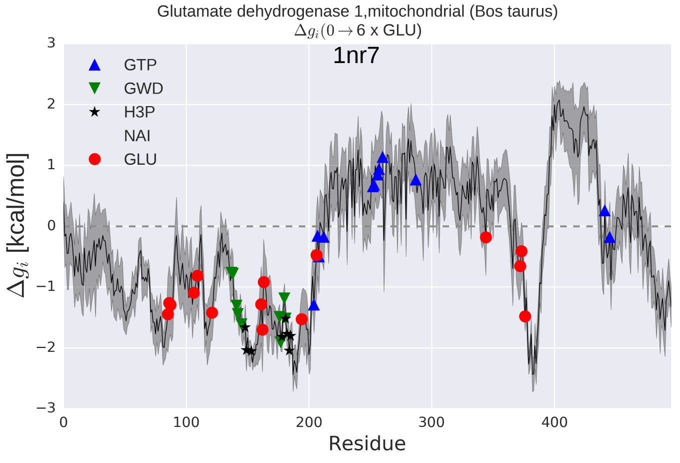
Glutamate dehydrogenase 1,mitochondrial (Bos taurus)
$\Delta g_i(0 \rightarrow 6$ x GLU$)$
1nr7
GTP
GWD
H3P
NAI
GLU
Δg_i [kcal/mol]
Residue

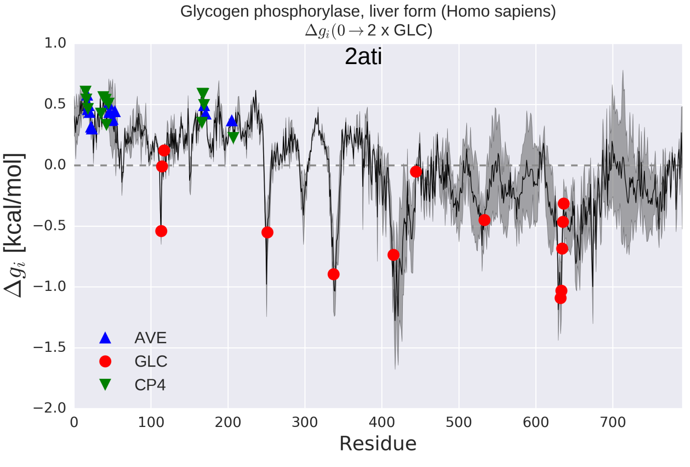
Glycogen phosphorylase, liver form (Homo sapiens)
$\Delta g_i(0 \rightarrow 2$ x GLC$)$
2ati
AVE
GLC
CP4
Δg_i [kcal/mol]
Residue

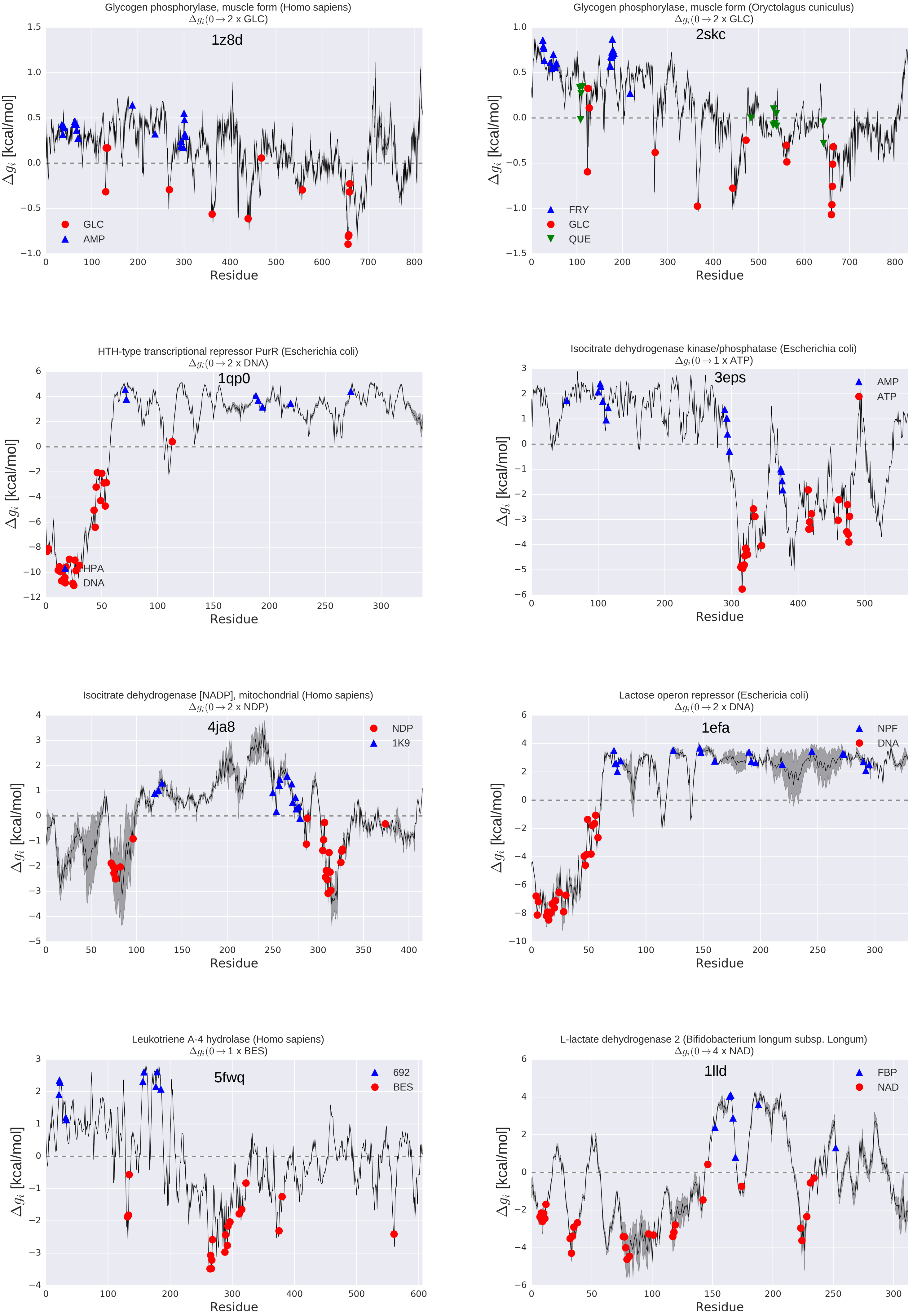
Glycogen phosphorylase, muscle form (Homo sapiens)
$\Delta g_i(0 \rightarrow 2$ x GLC)
1z8d
GLC
AMP
Δg_i [kcal/mol]
Residue
Glycogen phosphorylase, muscle form (Oryctolagus cuniculus)
$\Delta g_i(0 \rightarrow 2$ x GLC)
2skc
FRY
GLC
QUE
Δg_i [kcal/mol]
Residue
HTH-type transcriptional repressor PurR (Escherichia coli)
$\Delta g_i(0 \rightarrow 2$ x DNA)
1qp0
HPA
DNA
Δg_i [kcal/mol]
Residue
Isocitrate dehydrogenase kinase/phosphatase (Escherichia coli)
$\Delta g_i(0 \rightarrow 1$ x ATP)
3eps
AMP
ATP
Δg_i [kcal/mol]
Residue
Isocitrate dehydrogenase [NADP], mitochondrial (Homo sapiens)
$\Delta g_i(0 \rightarrow 2$ x NDP)
4ja8
NDP
1K9
Δg_i [kcal/mol]
Residue
Lactose operon repressor (Eschericia coli)
$\Delta g_i(0 \rightarrow 2$ x DNA)
1efa
NPF
DNA
Δg_i [kcal/mol]
Residue
Leukotriene A-4 hydrolase (Homo sapiens)
$\Delta g_i(0 \rightarrow 1$ x BES)
5fwq
692
BES
Δg_i [kcal/mol]
Residue
L-lactate dehydrogenase 2 (Bifidobacterium longum subsp. Longum)
$\Delta g_i(0 \rightarrow 4$ x NAD)
1lld
FBP
NAD
Δg_i [kcal/mol]
Residue

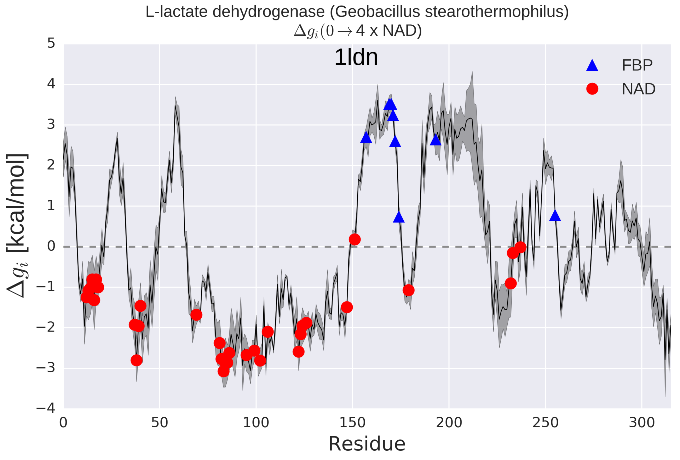
L-lactate dehydrogenase (Geobacillus stearothermophilus)
$\Delta g_i(0 \rightarrow 4$ x NAD)
1ldn
FBP
NAD
Δg_i [kcal/mol]
Residue

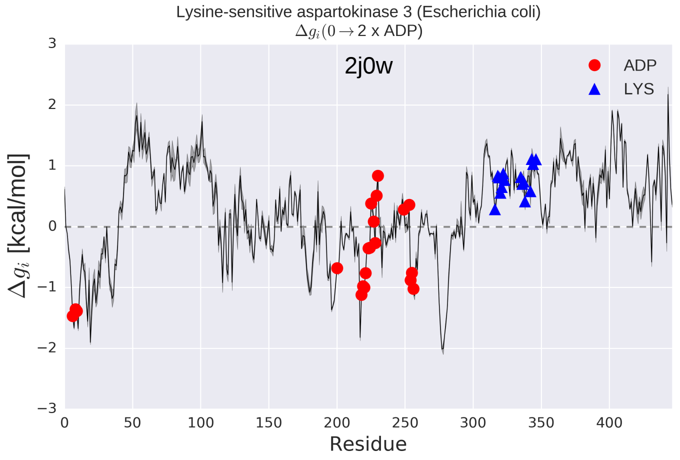
Lysine-sensitive aspartokinase 3 (Escherichia coli)
$\Delta g_i(0 \rightarrow 2$ x ADP)
2j0w
ADP
LYS
Δg_i [kcal/mol]
Residue

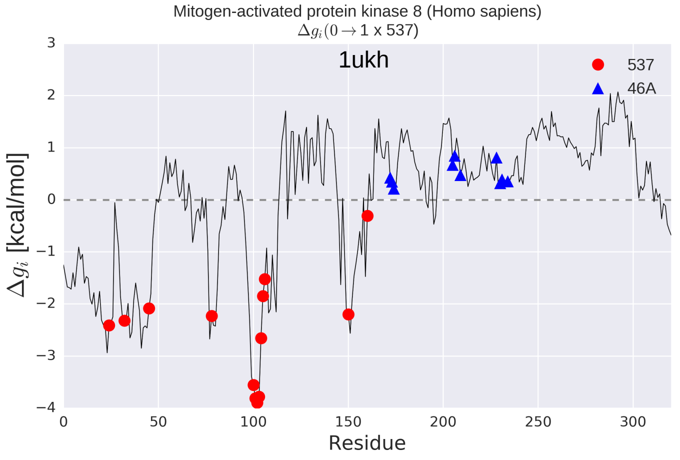
Mitogen-activated protein kinase 8 (Homo sapiens)
$\Delta g_i(0 \rightarrow 1$ x 537)
1ukh
537
46A
Δg_i [kcal/mol]
Residue

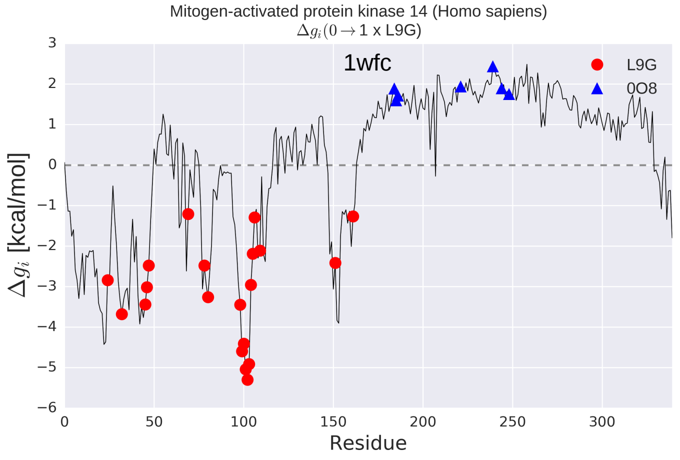
Mitogen-activated protein kinase 14 (Homo sapiens)
$\Delta g_i(0 \rightarrow 1$ x L9G)
1wfc
L9G
0O8
Δg_i [kcal/mol]
Residue

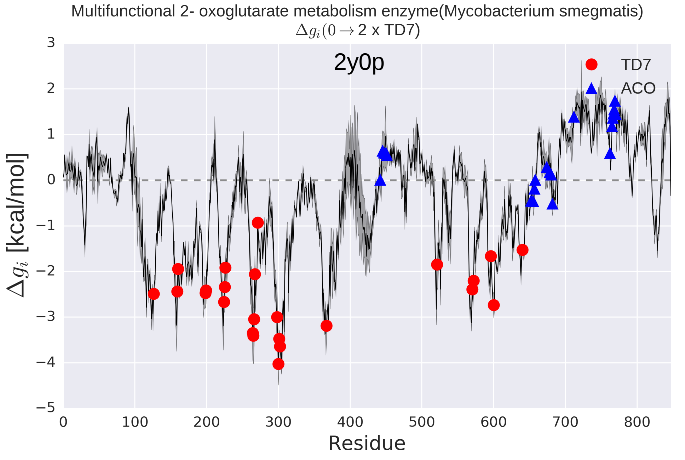
Multifunctional 2- oxoglutarate metabolism enzyme(Mycobacterium smegmatis)
$\Delta g_i(0 \rightarrow 2$ x TD7)
2y0p
TD7
ACO
Δg_i [kcal/mol]
Residue

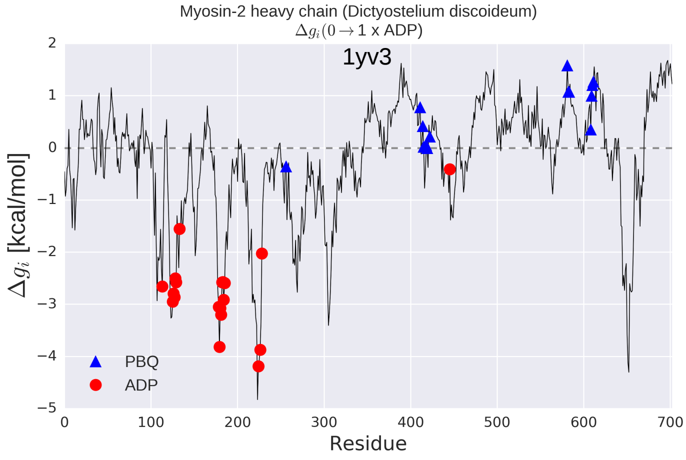
Myosin-2 heavy chain (Dictyostelium discoideum)
$\Delta g_i(0 \rightarrow 1$ x ADP)
1yv3
PBQ
ADP
Δg_i [kcal/mol]
Residue

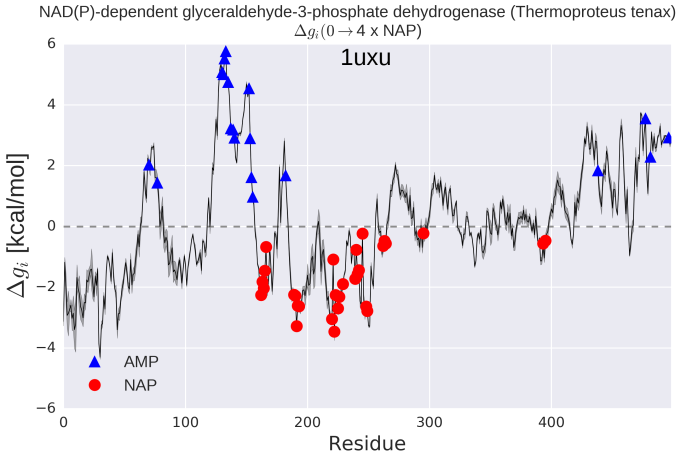
NAD(P)-dependent glyceraldehyde-3-phosphate dehydrogenase (Thermoproteus tenax)
$\Delta g_i(0 \rightarrow 4$ x NAP)
1uxu
AMP
NAP
Δg_i [kcal/mol]
Residue

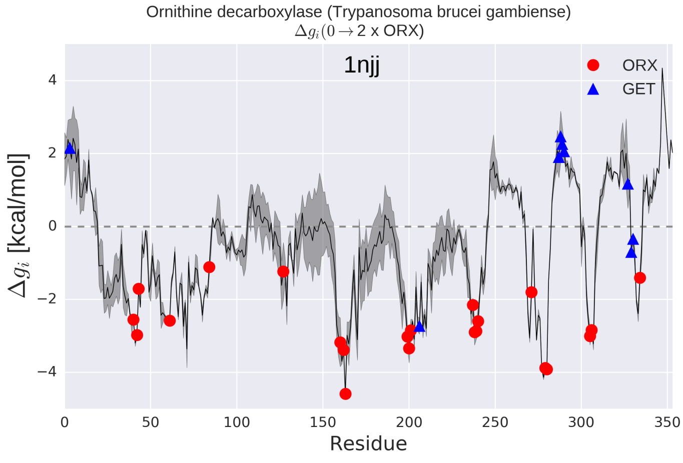
Ornithine decarboxylase (Trypanosoma brucei gambiense)
$\Delta g_i(0 \rightarrow 2$ x ORX)
1njj
ORX
GET
Δg_i [kcal/mol]
Residue

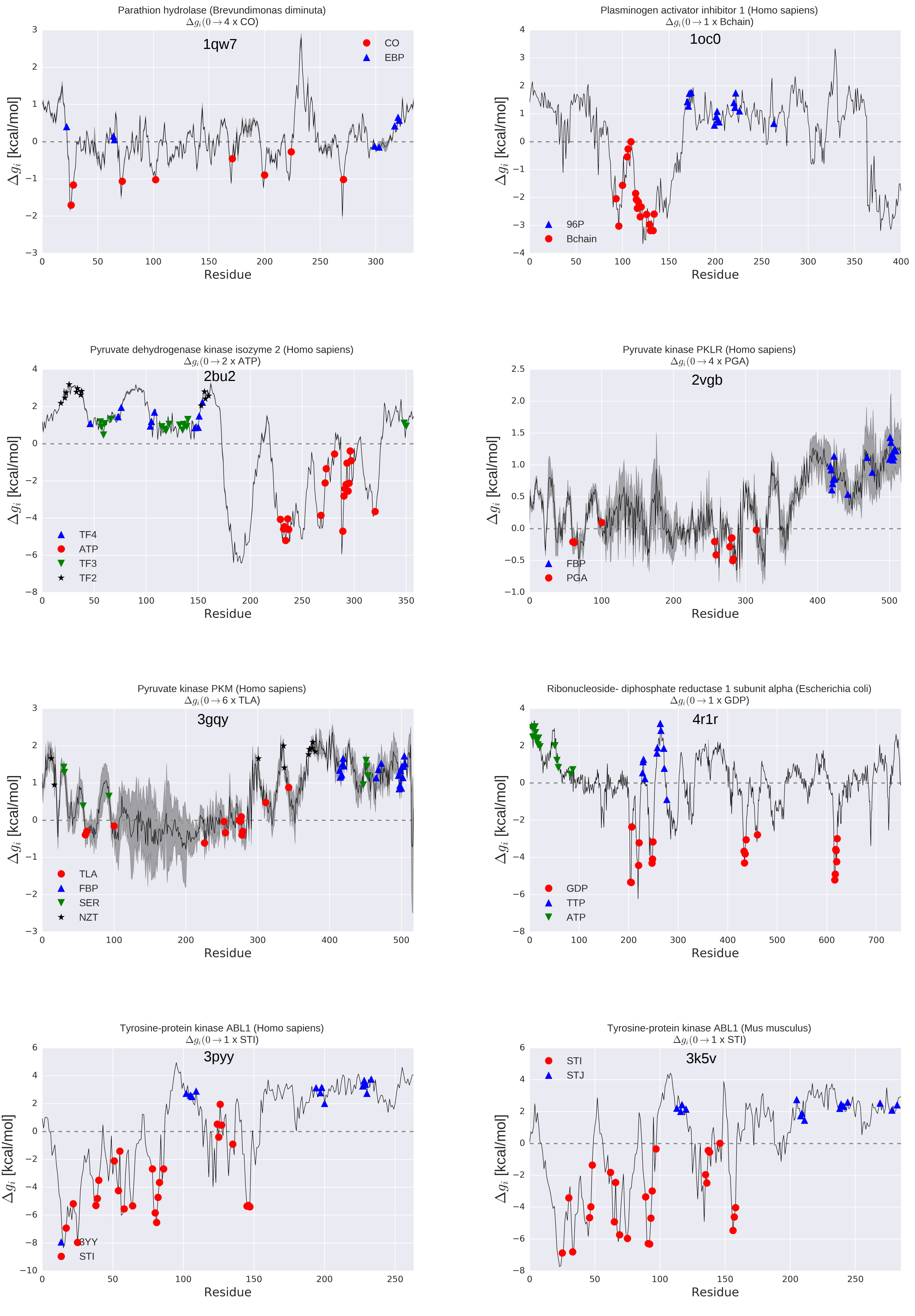
Parathion hydrolase (Brevundimonas diminuta)
$\Delta g_i(0 \rightarrow 4$ x CO)
1qw7
CO
EBP
Δg_i [kcal/mol]
Residue
Plasminogen activator inhibitor 1 (Homo sapiens)
$\Delta g_i(0 \rightarrow 1$ x Bchain)
1oc0
96P
Bchain
Δg_i [kcal/mol]
Residue
Pyruvate dehydrogenase kinase isozyme 2 (Homo sapiens)
$\Delta g_i(0 \rightarrow 2$ x ATP)
2bu2
TF4
ATP
TF3
TF2
Δg_i [kcal/mol]
Residue
Pyruvate kinase PKLR (Homo sapiens)
$\Delta g_i(0 \rightarrow 4$ x PGA)
2vgb
FBP
PGA
Δg_i [kcal/mol]
Residue
Pyruvate kinase PKM (Homo sapiens)
$\Delta g_i(0 \rightarrow 6$ x TLA)
3gqy
TLA
FBP
SER
NZT
Δg_i [kcal/mol]
Residue
Ribonucleoside- diphosphate reductase 1 subunit alpha (Escherichia coli)
$\Delta g_i(0 \rightarrow 1$ x GDP)
4r1r
GDP
TTP
ATP
Δg_i [kcal/mol]
Residue
Tyrosine-protein kinase ABL1 (Homo sapiens)
$\Delta g_i(0 \rightarrow 1$ x STI)
3pyy
3YY
STI
Δg_i [kcal/mol]
Residue
Tyrosine-protein kinase ABL1 (Mus musculus)
$\Delta g_i(0 \rightarrow 1$ x STI)
3k5v
STI
STJ
Δg_i [kcal/mol]
Residue

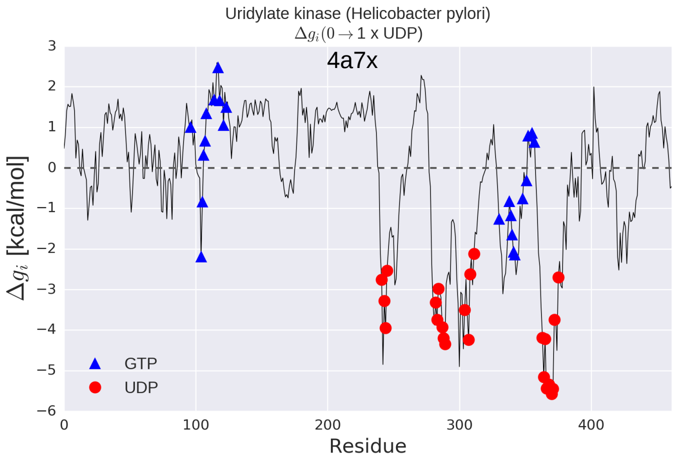

Uridylate kinase (Helicobacter pylori)
$\Delta g_i(0 \rightarrow 1$ x UDP)
4a7x
Δg_i [kcal/mol]
Residue
GTP
UDP
3
2
1
0
−1
−2
−3
−4
−5
−6
0
100
200
300
400
