# Supplementary material for: Reversing allosteric communication: From detecting allosteric sites to inducing and tuning targeted allosteric response
Source: PLoS Comput Biol. 2018 Jun 18;14(6):e1006228. doi: 10.1371/journal.pcbi.1006228 (PMC6023240; doi:10.1371/journal.pcbi.1006228)
Supplement: S1 Table — The list is obtained on the basis of the operational definition of allosteric sites. Predictive power for known allosteric sites in the protein, which is quantified by the area under the ROC curves (AUCs) for positive free energies, is given in the last column. (DOCX) [file pcbi.1006228.s008.docx]

| **Protein (species)** | **PDB** | **Restrained functional**  **site** | **Δ**$g_{F}$ **(kcal/mol)** | **Allosteric Site** | **Δ**$g_{A}$ **(kcal/mol)** | **Proximity (%)** | **AUC** |
| --- | --- | --- | --- | --- | --- | --- | --- |
| Acetyltransferase Pat (Mycobacterium tuberculosis) | 4avb | 1 x ACO | -4.92 | 1 x CMP | 3.74 | 0 | 0.67 |
| Amino-acid  acetyltransferase (Neisseria  gonorrhoeae) | 3d2p | 6 x COA | -1.14 | 6 x ARG | 1.21 | 0 | 0.41 |
| Anaerobic ribonucleoside-  triphosphate reductase (Enterobacteria phage T4) | 1h78 | 2 x DCF | -1.81 | 2 x DCP | 1.31 | 0 | 0.76 |
| Androgen receptor  (Homo sapiens) | 2pio | 1 x DHT | -4.61 | 1 x YLO | 0.74 | 0 | 0.70 |
| Androgen receptor  (Mus musculus) | 2qpy | 1 x DHT | -4.42 | 1 x 4HY | 2.0 | 0 | 0.71 |
| Antithrombin III  (Homo sapiens) | 1t1f | 1 x RCL | -4.62 | 1 x NTP | 0.96 | 0 | 0.72 |
| ATP phosphoribosyltransfe rase (Mycobacteriu  m tuberculosis) | 1nh8 | 2 x AMP | -1.59 | 2 x HIS | 0.97 | 0 | 0.69 |
| cAMP receptor protein (Mycobacterium tuberculosis) | 3d0s | 2 x DNA | -1.8 | 2 x CMP | 1.11 | 0 | 0.56 |
| Chorismate mutase (Saccharomyces  cerevisiae) | 3csm | 2 x TSA | -1.81 | 2 x TRP | 0.16 | 0.83 | 0.16 |
| Farnesyl pyrophosphate synthase (Homo sapiens) | 2qis | 2 x RIS | -1.38 | 4 x 3N1 | 0.44 | 0.63 | 0.47 |
| Fatty acid metabolism regulator protein (Escherichia coli) | 1hw1 | 2 x DNA | -5.44 | 2 x COA | 2.39 | 0 | 0.72 |
| Fructose-1,6- bisphosphatase 1 (Escherichia coli) | 2q8m | 4 x FBP | -0.77 | 4 x AMP  4 x BG6 | 1.67  -1.62 | 0  10 | 0.79  0 |
| Fructose-1,6- bisphosphatase 1 (Sus scrofa) | 1fbp | 4 x FBP | -1.01 | 4 x AMP  4 x PFE | 1.36  1.45 | 0  1.86 | 0.63  0.63 |
| Glucose-1-phosphate thymidylyltransferase 1 (Escherichia coli) | 1mc3 | 4 x TTP | 0.19 | 4 x TMP | 1.26 | 1.69 | 0.63 |
| Glutamate dehydrogenase 1,  mitochondrial (Bos taurus) | 1nr7 | 6 x GLU | -1.15 | 6 x GWD | -1.25 | 0 | 0.59 |
| Glycogen phosphorylase, liver  form (Homo sapiens) | 2ati | 2 x GLC | -0.66 | 2 x AVE  1 x CP4 | 0.45  0.5 | 0  0 | 0.79  0.89 |
| Glycogen phosphorylase, muscle form (Homo sapiens) | 1z8d | 2 x GLC | -0.5 | 2 x AMP | 0.4 | 0 | 0.69 |
| Glycogen phosphorylase, muscle form (Oryctolagus  cuniculus) | 2skc | 2 x GLC | -0.65 | 2 x FRY  2 x QUE | 0.67  0.02 | 0  0 | 0.88  0.24 |
| HTH-type transcriptional repressor PurR (Escherichia coli) | 1qp0 | 2 x DNA | -7.37 | 2 x HPA | 4.04 | 0 | 0.69 |
| Isocitrate dehydrogenase [NADP], mitochondrial (Homo sapiens) | 4ja8 | 2 x NDP | -1.6 | 1 x 1K9 | 0.95 | 0.32 | 0.5 |
| Isocitrate dehydrogenase kinase/phosphatase (Escherichia coli) | 3eps | 1 x ATP | -3.58 | 1 x AMP | 0.67 | 0.28 | 0.51 |
| L-lactate dehydrogenase (Geobacillus stearothermophilus) | 1ldn | 4 x NAD | -1.85 | 2 x FBP | 2.89 | 0.41 | 0.79 |
| L-lactate dehydrogenase 2 (Bifidobacterium longum subsp. Longum) | 1lld | 4 x NAD | -2.78 | 2 x FBP | 3.26 | 0.52 | 0.78 |
| Lactose operon repressor (Eschericia coli) | 1efa | 2 x DNA | -5.97 | 2 x NPF | 3.16 | 0 | 0.64 |
| Leukotriene A-4 hydrolase (Homo sapiens) | 5fwq | 1 x BES | -2.47 | 1 x 692 | 2.08 | 0 | 0.87 |
| Lysine-sensitive  aspartokinase 3 (Escherichia coli) | 2j0w | 2 x ADP | -0.56 | 2 x LYS | 0.74 | 0 | 0.53 |
| Mitogen-activated protein kinase 14 (Homo sapiens) | 1wfc | 1 x L9G | -3.48 | 1 x 0O8 | 1.88 | 0 | 0.8 |
| Mitogen-activated  protein kinase 8 (Homo sapiens) | 1ukh | 1 x 537 | -2.92 | 1 x 46A | 0.82 | 0 | 0.36 |
| Multifunctional 2- oxoglutarate  metabolism enzyme (Mycobacterium  smegmatis) | 2y0p | 2 x TD7 | -2.85 | 2 x ACO | 0.62 | 0 | 0.61 |
| Myosin-2 heavy chain (Dictyostelium  discoideum) | 1yv3 | 1 x ADP | -2.94 | 1 x PBQ | 0.58 | 0 | 0.58 |
| NAD(P)-dependent glyceraldehyde-3- phosphate  dehydrogenase (Thermoproteus  tenax) | 1uxu | 4 x NAP | -1.93 | 4 x AMP | 3.52 | 0 | 0.84 |
| Ornithine decarboxylase (Trypanosoma brucei gambiense) | 1njj | 2 x ORX | -2.87 | 2 x GET | 1.23 | 0.77 | 0.8 |
| Parathion hydrolase (Brevundimonas diminuta) | 1qw7 | 4 x CO | -1.42 | 2 x EBP | 0.22 | 0 | 0.33 |
| Plasminogen activator inhibitor 1 (Homo sapiens) | 1oc0 | 1 x Bchain | -2.19 | 1 x 96P | 1.09 | 0 | 0.47 |
| Pyruvate dehydrogenase kinase  isozyme 2 (Homo sapiens) | 2bu2 | 2 x ATP | -3.3 | 2 x TF2  2 x TF3  2 x TF4 | 2.67  0.85  1.14 | 0  0.94  0 | 0.85  0.22  0.34 |
| Pyruvate kinase PKLR (Homo sapiens) | 2vgb | 4 x PGA | -0.13 | 4 x FBP | 1.05 | 0 | 0.75 |
| Pyruvate kinase PKM (Homo sapiens) | 3gqy | 6 x TLA | -0.22 | 4 x FBP  2 x NZT  4 x SER | 1.25  1.8  1.22 | 0  0  1.58 | 0.59  0.83  0.58 |
| Ribonucleoside- diphosphate reductase 1 subunit alpha (Escherichia coli) | 4r1r | 1 x GDP | -4.39 | 1 x ATP  1 x TTP | 2.12  1.28 | 0  0 | 0.79  0.69 |
| Tyrosine-protein kinase ABL1 (Homo sapiens) | 3pyy | 1 x STI | -4.13 | 1 x 3YY | 2.93 | 0 | 0.58 |
| Tyrosine-protein  kinase ABL1 (Mus musculus) | 3k5v | 1 x STI | -4.56 | 1 x STJ | 2.38 | 0 | 0.54 |
| Uridylate kinase (Helicobacter pylori) | 4a7x | 1 x UDP | -4.24 | 2 x GTP | 0.05 | 1.82 | 0.54 |
